# The application of nanotechnology in regulating mitochondrial function in tumor microenvironment for cancer therapy

**DOI:** 10.7150/thno.121956

**Published:** 2026-01-01

**Authors:** Dazhong Wang, Meng Yuan, Ji Liu, Ming Zhao, Ting Fang

**Affiliations:** 1 Department of Pharmaceutics, School of Pharmacy, Shenyang Pharmaceutical University, No. 103, Wenhua Road, Shenyang, 110016, P. R. China.; 2 Cancer Hospital of Dalian University of Technology, Liaoning Cancer Hospital & Institute No. 44 Xiaoheyan Road, Dadong District, Shenyang, 110042, Liaoning Province, P. R. China.

**Keywords:** tumor treatment, tumor microenvironment, mitochondrial function, nanodrugs

## Abstract

Mitochondria are involved in energy production, signal conduction, and cellular differentiation in the human body, and they determine the direction of tumorigenesis and development. Mitochondria-targeted therapy in cancer cells has been reported since researchers discovered the relationship between mitochondria and cancer. However, the complexity of the tumor microenvironment (TME) can impair the therapeutic effect. Understanding the mechanisms of mitochondrial function in various cells of TME (e.g., tumor-associated macrophages (TAMs), cancer-associated fibroblasts (CAFs), cancer stem cells (CSCs), T cells, natural killer (NK) cells, tumor-associated neutrophils (TANs)), as well as mediated crosstalk with cancer cells, would be beneficial for accelerating these therapeutic strategies into clinical practice and leading to more effective disease treatment. Subsequently, we summarized representative small-molecule drugs targeting mitochondrial homeostasis, energy metabolism, and mitochondrial DNA (mtDNA) and evaluated their limitations. Building on this foundation, we reviewed the latest multifunctional nanomedicines. These agents leverage TME responsiveness, surface-targeting engineering, and multimodal synergy (combining chemotherapy, photodynamic therapy (PDT), sonodynamic therapy (SDT), radiodynamic therapy (RDT), and immunotherapy) to precisely deliver drugs, ions, genetic material, and even whole mitochondria to target organelles. This approach simultaneously remodels the immunosuppressive microenvironment and induces immunogenic cell death (ICD).

## 1. Introduction

There are several leading causes of cancer in all countries, as well as a number of barriers to increasing life expectancy because of cancer 1. Current treatments for tumors include surgery, chemotherapeutic drugs, radiotherapy, and immunotherapy, administered alone or in combination. However, issues are emerging, such as multidrug resistance (MDR), stem-like cell growth, and an immunosuppressive tumor microenvironment (TME), due to the non-specific targeting of tumor cells and low cytotoxicity 2, 3. Mitochondria have long been recognized as the metabolic engines of eukaryotic cells, and in the context of cancer, they operate as decision-making hubs that integrate nutrient availability, redox status, and death-survival signals 4-6. Mitochondria are crucial for cancer cell survival through mechanisms like morphology, mitophagy, mitochondrial reactive oxygen species (mtROS) production, metabolism, mitochondrial DNA (mtDNA), and calcium signaling. They are essential for tumor development, invasion, metastasis, and treatment resistance, and also regulate cell growth, differentiation, and apoptosis 5.

Mitochondria orchestrate oncogenesis through multiple, interconnected mechanisms. Dynamic cycles of fusion and fission dictate metabolic flexibility, allowing tumors to switch between glycolysis and oxidative phosphorylation (OXPHOS) in response to fluctuating nutrient and oxygen availability 7-9. Excessive mtROS generated by a hyperactive electron transport chain (ETC) promotes genomic instability, epithelial-mesenchymal transition (EMT), and therapy resistance 10. Importantly, these adaptations are not restricted to malignant cells; tumor-associated macrophages (TAMs), cancer-associated fibroblasts (CAFs), cancer stem cells (CSCs), tumor-infiltrating T cells (TILs), natural killer (NK) cells, and neutrophils all exhibit mitochondria-driven metabolic identities that collectively shape an immunosuppressive and metabolically hostile niche 11. Emerging research highlights intercellular mitochondrial transfer as a critical focus, demonstrating that: tumor cells deliver mitochondria harboring mutated mtDNA to TILs, impairing their antitumor functionality 12; mitochondrial transfer from CAFs to tumor cells promotes metastatic progression 13; while pharmacologically blocking mitochondrial trafficking from immune cells to tumor cells effectively suppresses tumor growth in experimental models 14.

Strategies targeting mitochondrial modulation offer a promising solution. The high negative membrane potential of the mitochondrial inner membrane necessitates unique physicochemical properties for drug delivery 15. Lipophilic cations exploit electrostatic attraction and hydrophobic interactions to accumulate within the mitochondrial matrix, with triphenylphosphonium (TPP) and dequalinium (DQA) serving as widely exploited mitochondrial-targeting moieties for small molecule design 16. Although exhibiting high mitochondrial specificity, these cationic carriers frequently induce multidrug resistance, compromising therapeutic efficacy 17. Although compounds like the Bcl-2 antagonist ABT-737 promote tumor apoptosis by altering mitochondrial membrane permeability, their therapeutic potential is limited by challenges such as poor oral bioavailability 18. Biguanide antidiabetic drugs (e.g., metformin, phenformin, buformin), established mitochondrial ETC inhibitors, have yielded disappointing clinical outcomes to date 19. Notably, IACS-010759 effectively suppresses proliferation in glycolysis-deficient hypoxic tumor cells. Paradoxically, OXPHOS inhibition by this compound induces mitochondrial transfer from bone marrow mesenchymal stem cells (MSCs) to acute myeloid leukemia (AML) cells via tunneling nanotubes (TNTs), while concurrently triggering mitochondrial fission and mitophagy in AML cells—mechanisms that collectively enhance cancer cell migration 20.

The complexity of the TME, however, has hindered the clinical translation of conventional small-molecule mitochondrial modulators 21. Systemic administration often leads to off-target toxicities in highly metabolic organs, while heterogeneous intra-tumoral oxygenation gradients and enzymatic heterogeneity establish pharmacological sanctuaries that facilitate resistance development 22. Nanotechnology offers an unprecedented opportunity to overcome these barriers. By exploiting pH gradients, enzymatic signatures, or receptor overexpression within the TME, nanodrug delivery systems (NDDS) can deliver drugs, metal complexes, nucleic acids, or even isolated mitochondria to defined subcellular locations 16, 23. Furthermore, precise modulation of mitochondrial dysfunction can be achieved through either monotherapy or combination regimens incorporating chemotherapy, photodynamic therapy (PDT), sonodynamic therapy (SDT), radiodynamic therapy (RDT), and/or immunotherapy 24, 25; concurrently inducing immunogenic cell death (ICD) characterized by damage-associated molecular pattern release, thereby initiating adaptive antitumor immunity 26.

In this review, we synthesize the latest advances in understanding mitochondria-mediated crosstalk between tumor cells and their microenvironmental partners. First, we dissect the metabolic reprogramming of each major TME component, highlighting how mitochondrial dynamics, biogenesis, mitophagy, and metabolite exchange dictate pro- or anti-tumor phenotypes. Second, we catalog emerging pharmacological agents that target mitochondrial balance, energy metabolism, or mtDNA integrity, assessing their therapeutic potential and limitations. Finally, we critically evaluate how multifunctional nanodrugs can be engineered to translate these insights into precision cancer therapy, and we outline the key translational hurdles that must be addressed to bring mitochondria-targeted nanomedicine to the clinic.

## 2. Mitochondria-Mediated Cellular Crosstalk within TME

**Figure 1:**
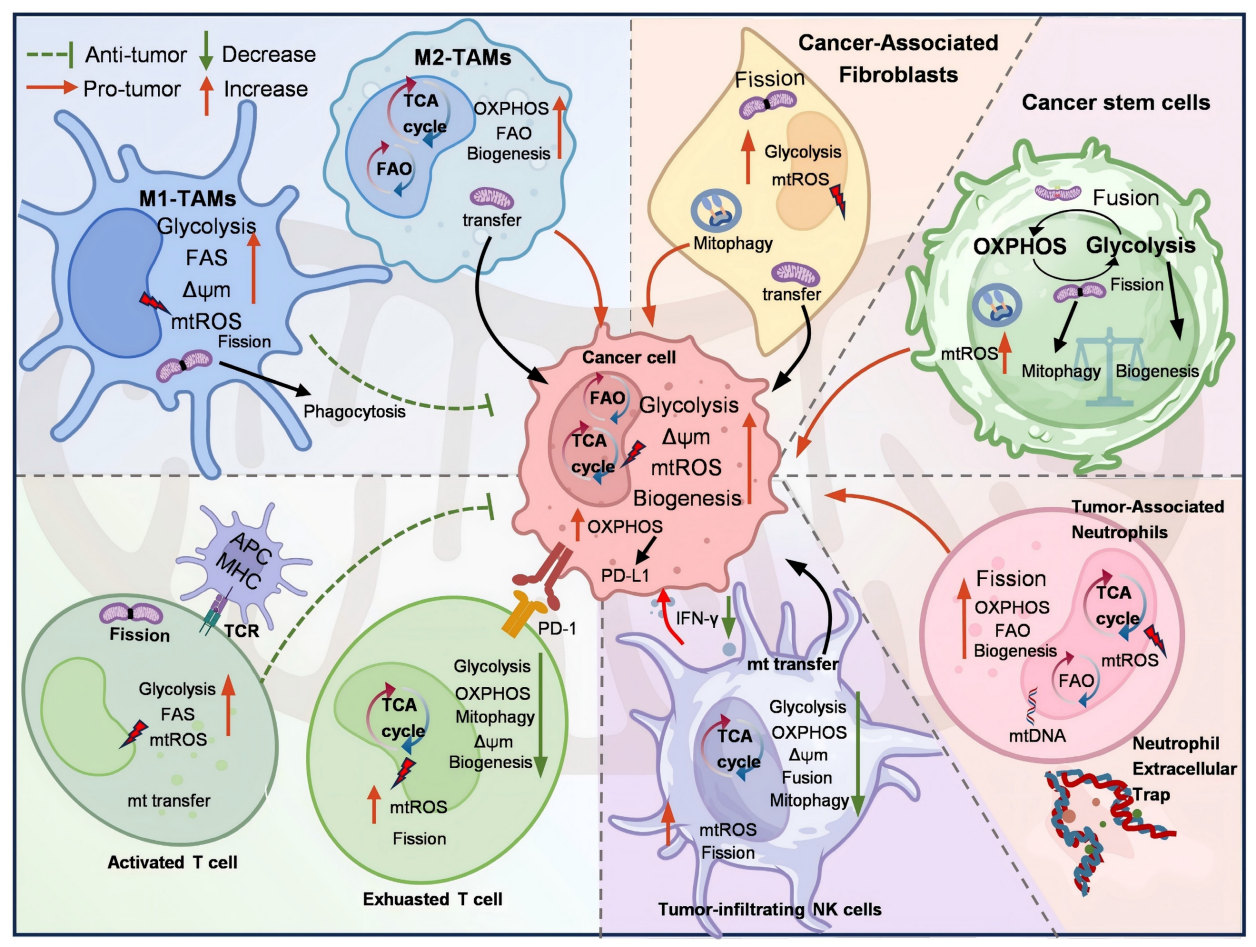
The role of mitochondria in TME. Mitochondria-mediated crosstalk between cancer cells and cancer-associated cells within TME, inducing the mitochondrial metabolic reprogramming and anti-/pro-tumor effect. Created with BioRender.com. (http://biorender.com).

Mitochondria in cancer cells are different from those in normal cells in terms of energy metabolism, increased mitochondrial membrane potential (MMP), ROS production, low oxygen (O_2_) consumption, and high glutathione (GSH) levels 23. Similarly, the mitochondria of tumor-associated stromal cells, CSCs, and tumor-associated immune cells are also different from those of normal cells, promoting or inhibiting tumor progression. This section aims to investigate the mechanism of mitochondria-mediated crosstalk between cancer cells and various cancer-associated cells, including TAMs, CAFs, CSCs, lymphoid lineage immune cells, and TANs within the TME (Figure 1).

### 2.1 Mitochondria-mediated Interactions between TAMs and tumor cells

TAMs, defined as macrophages (MФ) that infiltrate and reside within TMEs, can be categorized into two distinct subsets. M1-TAMs are induced by T helper 1 (Th1) cytokines, including interferon-gamma (IFN-γ), granulocyte-macrophage colony-stimulating factor, and lipopolysaccharide. Conversely, M2-TAMs are activated by T helper 2 (Th2) cytokines, such as interleukin-4 (IL-4), interleukin-10 (IL-10), macrophage colony-stimulating factor (M-CSF), or tumor cell-surface molecules 27-29. Notably, TAMs with an anti-inflammatory and pro-tumor M2 phenotype predominantly secrete immunosuppressive cytokines, such as IL-10 and transforming growth factor-beta (TGF-β), thereby promoting tumor proliferation and metastasis. In contrast, pro-inflammatory and anti-tumor M1-TAMs inhibit tumor progression and induce tissue damage by secreting IL-6, tumor necrosis factor-alpha (TNF-α), nitric oxide (NO), and ROS 30.

**Figure 2:**
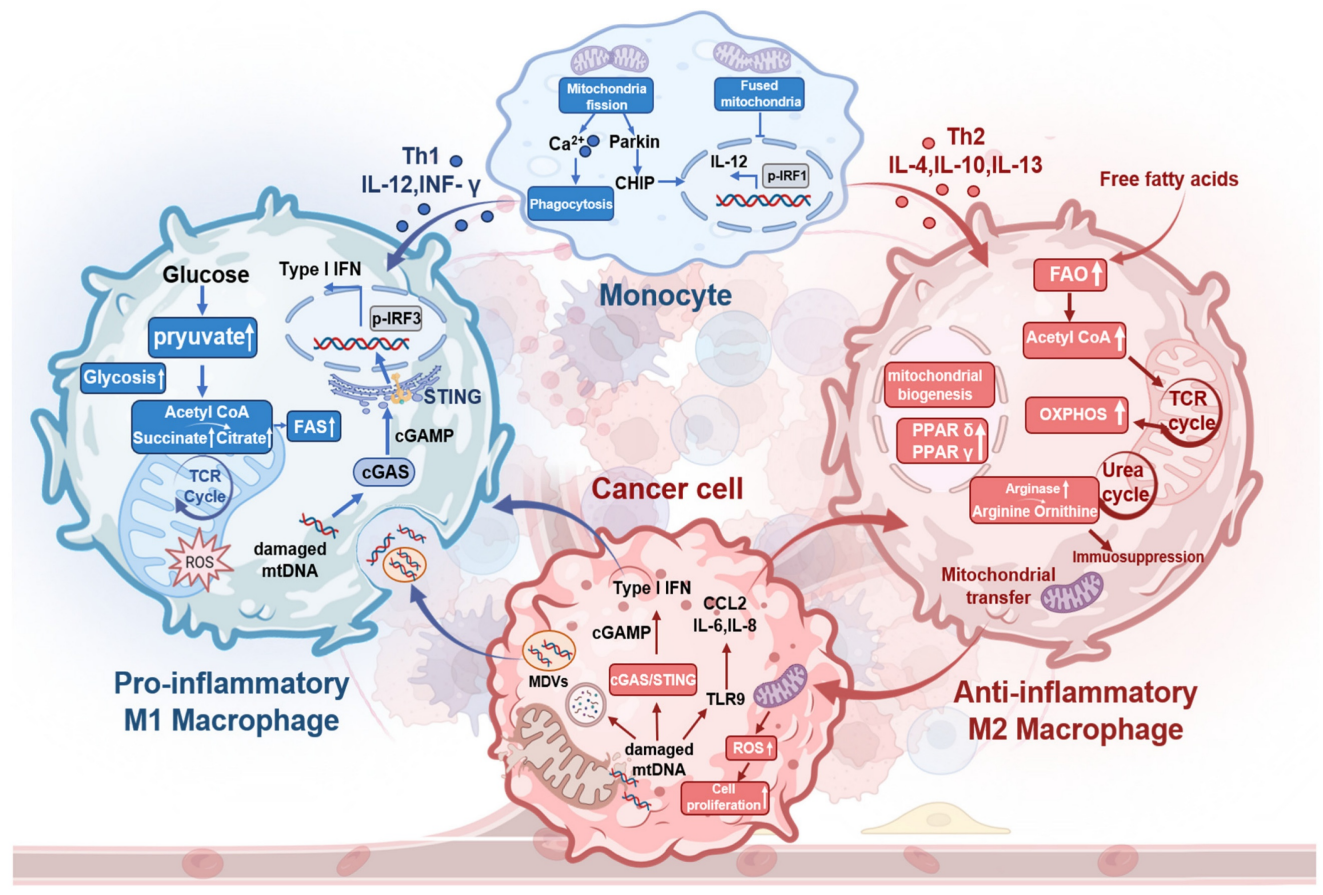
Mitochondria metabolic reprogramming within cancer cells and TAMs and mitochondria-mediated crosstalk between them. Macrophage mitochondrial fission enhances phagocytosis, while fusion suppresses it. Beyond induction by Th1 cytokines, tumor-released damaged mtDNA activates cGAS-STING to drive IFN-I-mediated M1-TAM polarization and stimulates TLR9 to promote M2-TAM polarization. M1-TAMs shift from OXPHOS to glycolysis, increasing pyruvate flux toward acetyl-CoA; subsequent TCA cycle elevation boosts citrate (for FAS) and succinate (for ROS generation). Conversely, M2-TAMs exhibit increased free fatty acid influx fueling FAO, which enhances TCA cycling, OXPHOS, and mitochondrial biogenesis. Mitochondrial transfer from M2-TAMs to tumor cells induces ROS bursts that promote tumor proliferation. Created with BioRender.com. (http://biorender.com).

M1-TAMs predominantly use glycolysis for metabolism, increasing citrate and succinate levels. These metabolites fuel FAS and boost mitochondrial ROS and mtDNA production 31. mtDNA activates the cGAS-STING pathway in TAMs, triggering IFN-I secretion (Figure 2). In contrast, IL-4 activates M2-TAMs, allowing FFA influx that stimulates STAT6. This promotes FAO, mitochondrial biogenesis, and TCA cycle activity to generate ATP, NADH, and FADH2 for OXPHOS 32. M2-TAMs also upregulate ARG1, ARG2, and IDO1, diverting L-arginine to the urea cycle. These key metabolic changes in TME promote TGF-β secretion, thereby suppressing effector T cells. Additionally, TAM mitochondrial reprogramming releases cytokines (CCL2, IL-1α, IL-6, TNF-α) to effectively support colon cancer growth 33. Activated STAT6 drives PPARδ and PPARγ expression, leading to the overexpression of anti-inflammatory genes.

A continuous mitochondrial network maintained via fusion sustains high FAO and OXPHOS levels in M2-TAMs. Altering TAM mitochondrial dynamics impacts tumor biology: fusion suppresses M1-TAM polarization by inhibiting IRF1-mediated IL-12 production, while fragmentation activates downstream IRF1 through Parkin-dependent CHIP proteolysis 34. Mitochondrial fission in MФ raises cytoplasmic calcium, enhancing phagocytosis via PKC-θ-mediated phosphorylation of WIP 35. Fragmented MФ overexpresses ARG1 and shows increased phagocytic activity. M1-TAMs feature elongated mitochondria compared to M2 and unpolarized MФ, but tumors inhibit TAM fission to evade clearance. TAM-to-cancer cell mitochondrial transfer via tunneling nanotubes promotes cell motility, invasion, and EMT 36, 37. This TAM-tumor mitochondrial crosstalk drives poor treatment outcomes.

### 2.2 Mitochondria-mediated Interactions between CAFs and tumor cells

CAFs are a heterogeneous population with different functions, mainly derived from resident fibroblasts that are induced by a variety of factors, such as TGF-β, IL-1β, and fibroblast growth factor (FGF). Mutual communication between CAFs and cancer cells involves the exchange of metabolites, the regulation of signaling pathways, and effects on tumor growth and metastasis. The activity of fibroblast mitochondria, which is involved in signaling pathways leading to metabolic switching and is often seen in CAFs, could either be a consequence of their activation by cancer cells or an active phenomenon favoring tumor development 38.

ROS and soluble factors (e.g., TGF-β) released by cancer cells mediate the transformation of normal fibroblasts into catabolic CAFs with reprogrammed metabolism from OXPHOS to aerobic glycolysis 39. Overproduction of ROS not only damages the cells but also induces mitophagy in the mitochondria of CAFs 40. Autophagy in CAFs inhibits tumor progression by promoting immunity and suppressing hypoxic conditions in the early stages. However, autophagy in CAFs promotes tumor development via providing nutrients (L-lactate, ketone bodies), cytokines (IL-1β, IL-6, IL-8), CXCL12, and α-SMA in advanced stages of tumorigenesis 41. Mitochondrial transfer from normal oral fibroblasts (NOFs) to OSCC via TNTs is similar to what occurs between TAMs and cancer cells 40.

CAFs provide intermediate metabolites for mitochondrial activity in cancer cells. These metabolites reorganize cancer cells' metabolism to fuel the TCA cycle and/or to promote mitochondrial biogenesis, as well as supply three major nutrients, thereby providing resources for tumor growth and metastasis. In addition to substance transport, and accumulation of mitochondrial metabolites in tumors can induce the upregulation of mitochondrial activity in cancer cells 42. Accumulation of succinate and fumarate induced HIF-1α stabilization and EMT, respectively 43. When co-cultured with OSCC cells, NOFs and CAFs are the main sources of L-lactate, which is transported across membranes via monocarboxylate transporters 44. The uptake of lactate by OSCC and prostate cancer cells shifts the NAD⁺/NADH ratio, leading to NADH accumulation. Subsequent oxidation of NADH by mitochondrial complex I drives ATP production, which fuels tumorigenic progression. Reciprocal interactions between CAFs and cancer cells have been shown to enhance the glycolytic capacity of CAFs, while cancer cells increase their mitochondrial function. Accumulation of mitochondrial metabolites induces EMT, disrupts redox balance, allowing for increased energy production by cancer cells, and stimulates tumor metastasis. The intricate dialogue between CAFs and cancer cells is a pivotal aspect of tumor progression and microenvironment modulation. In addition to the differences among these cancers, which depend on the tissues from which they originate, intra-tumor heterogeneity may be the source of interactions between different cancer cells and CAFs.

### 2.3 The mitochondria of CSCs

Treatment resistance in tumor cells and CSCs is a significant contributor to treatment failure and recurrence. CSCs, also termed as tumor-initiating cells, exhibit characteristics of tumor initiation, self-renewal, multidirectional differentiation, and plasticity within the TME 45, 46. Maintenance of stemness and differentiation of CSCs depends on mitochondrial regulation, including mitochondrial dynamics, mitochondrial metabolic reprogramming, homeostatic imbalance, and mtDNA depletion 47, 48.

**Figure 3:**
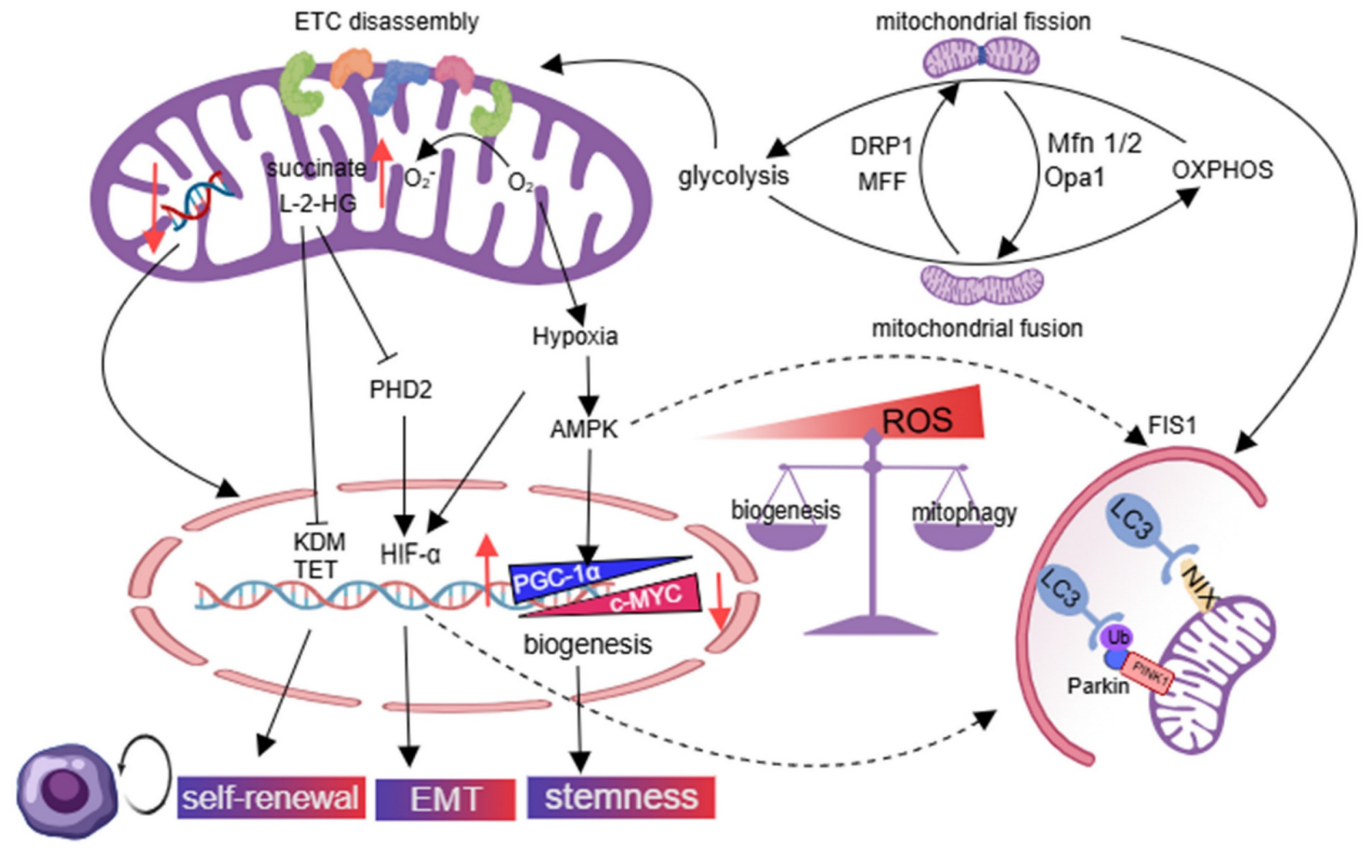
Mitochondrial metabolic reprogramming and homogeneity balance are disrupted in CSCs. Mitochondrial hyper-fission in cancer shifts metabolism from OXPHOS to glycolysis. Resultant mitochondrial dysfunction promotes CSC self-renewal, EMT, and stemness via altered nuclear proteins. Impaired mitochondrial biogenesis sustains CSCs through low ROS, while AMPK-activated mitophagy in LSCs elevates ROS to drive proliferation. Conversely, fusion-enriched tumors favor OXPHOS as a novel CSC trait. Created with Medpeer.com. (https://www.medpeer.cn).

Glycolysis can serve as the main energy source for stem cells, which helps CSCs survive under hypoxic conditions 49. The fragmented mitochondria are associated with overactivation of DRP1 and mitochondrial fission factor (MFF), which promote a metabolic transition from OXPHOS to glycolysis and attenuate ROS production in tamoxifen-resistant breast cancer, brain tumor-initiating cells, and liver CSCs, thereby preserving the self-renewal potential of CSCs 50. The compromised aerobic oxidation function of fragmented mitochondria leads to the buildup of succinate and L-2-hydroxyglutarate (L-2-HG), which impedes the activity of ten-eleven translocation and histone lysine demethylase enzymes, ultimately promoting the self-renewal capacity of CSCs 51. Succinate inhibits hydroxylation and subsequent degradation of HIF-1α by blocking α-ketoglutarate-dependent prolyl hydroxylases, and triggers the EMT, resulting in the emergence of CSCs. CSCs use mitophagy and mitochondrial biogenesis to maintain aggressive phenotypes and promote tumor relapse. AMPK/AP-1 pathway phosphorylates peroxisome proliferator-activated receptor γ co-activator 1α (PGC-1α), leading to improper mitochondria biogenesis, which supports the stemness of CSCs and their capacity for carcinogenesis 52, 53. The MYC/PGC-1α ratio determines metabolic phenotype in pancreatic CSCs, with MYC promoting a glycolytic phenotype and inhibiting OXPHOS, while PGC-1α promotes mitochondrial biogenesis. PGC-1α, with its antioxidant properties, maintains CSC self-renewal by controlling low ROS levels, while increasing ROS levels can induce CSC death 54. Fragmented mitochondria trigger mitophagy through BNIP3L in CSCs with glycolytic phenotypes, while adenosine monophosphate-activated protein kinase (AMPK) activation induces mitochondrial fission 1 (FIS1)-mediated mitophagy in leukemia stem cells (LSCs) to increase ROS levels and promote cell proliferation 55, 56. The balance between mitophagy and mitochondrial biogenesis is controlled by CSCs, promoting aggressive CSC phenotypes and tumor relapse. CSCs in leukemia, colon cancer, and cholangiocarcinoma prefer OXPHOS 45, 57, 58. Overexpression of mitochondrial fusion proteins (MFN1/2) and optic atrophy 1 (OPA1) upregulates mitochondrial mass, as well as enhances OXPHOS and NAD^+^ levels. Decreasing mtDNA copy number triggers retrograde signaling, leading to changes in nuclear gene expression that help cancer cells adapt to the tumor microenvironment and maintain their stemness 59 (Figure 3).

### 2.4 The mitochondria of T and B lymphocytes

**Figure 4:**
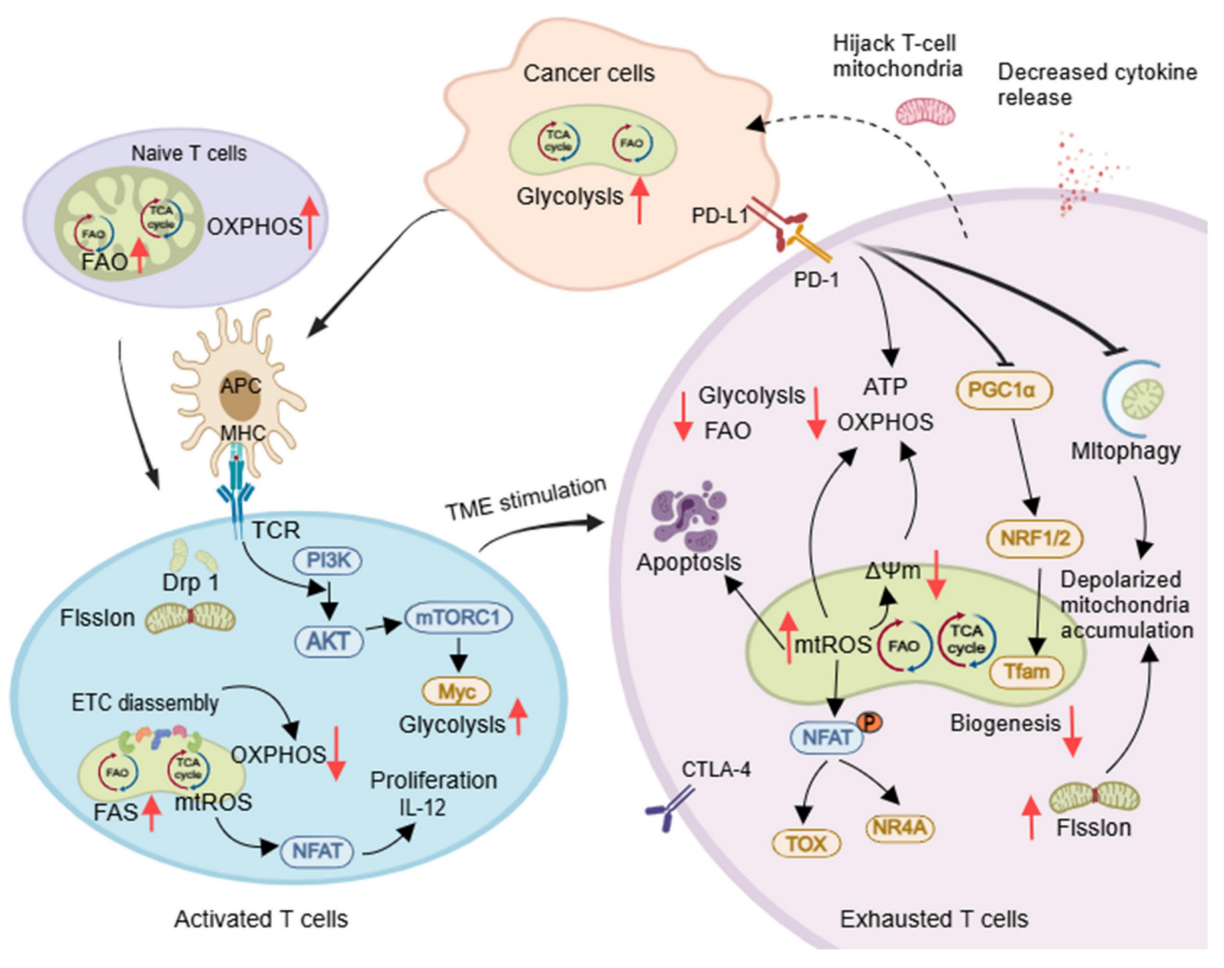
Mitochondria control the developmental and differentiation state of T cells. Naïve T cells primarily rely on OXPHOS and FAO for energy. Upon activation by APCs, T cells undergo Drp1-dependent mitochondrial fission and shift their metabolism toward aerobic glycolysis. However, within tumor microenvironments, exhausted T cells (TEX) exhibit high PD-1 expression. This suppresses OXPHOS, increases oxidative stress, and impairs mitochondrial biogenesis and function. Reduced mitophagy leads to accumulated depolarized mitochondria, while mitochondrial transfer further compromises T cell migration and activation - collectively accelerating TEX development. Created with Medpeer.com. (https://www.medpeer.cn).

The T cell population includes naïve (TN), effector (TE), memory (TM), and regulatory (Treg) cells. Mitochondrial structure and function change during T cell development, influencing their function. TN cells have round mitochondria that depend on OXPHOS and FAO for metabolic quiescence, while TE cells have fragmented mitochondria with increased glycolysis, FAS, and OXPHOS linked to their proliferative state 60 (Figure 4). Asymmetric division leads to TM/Treg cells, with TM cells showing long, tubular mitochondria through Opa1-dependent fusion. Memory T cells revert to a quiescent metabolic state supported by FAO and OXPHOS 61. CD8^+^ T cells with low mitochondrial membrane potential (ΔΨm) are more effective *in vivo* 62. Tregs, which inhibit T cell activation and proliferation, prefer the FAO pathway for energy efficiency 63.

The activation of expansion of T cells is supported by DRP1-dependent mitochondrial fission, which promotes aerobic glycolysis and increases mitochondrial numbers and cristae loosening. This process occurs at immune synapses between the T-cell receptor (TCR) and antigen-presenting cells (APC) 64. TCR also enhances glycolysis both in activated B lymphocytes [65]and T cells [66]via PI3K/AKT/mTOR pathway, and generates mtROS to activate the expression of transcription factor nuclear factor of activated T cells and IL-2, essential for maturation and proliferation of T cells 67. Mitochondrial cristae loosening can reduce ETC supercomplex efficiency, promoting aerobic glycolysis 68. Activated CD8^+^ T cells release perforin and granzyme and induce apoptosis mediated by death ligands/death receptors, leading to direct destruction of tumor cells (Figure 4). Defective mitochondrial fission in CD8^+^ T cells can reduce infiltration in solid cancer models 69.

T exhausted cells (TEX) within tumors are no longer able to respond to stimuli, and the onset of their exhaustion is driven by multiple tumor microenvironmental factors. These include persistent tumor antigenic stimulation, nutrient deprivation (such as glucose competition), metabolite accumulation (e.g., lactate), and hypoxia, as well as high expression of inhibitory receptors such as programmed cell death protein 1 (PD-1), TIM-3, LAG-3, and CTLA4 70, 71. These factors decrease mitochondrial quantity and alter function, restricting glycolysis due to glucose deficiency. Therapeutic strategies for TEX involve reducing ROS, lowering tumor hypoxia to prevent T cell exhaustion 71, inhibiting glycolysis to enhance CD8^+^ TM cell function 72, and promoting FAO to regulate mitochondrial CD8^+^ TILs (e.g., PGC-1α agonist bezafibrate) 73. Further research is needed to understand how tumor-infiltrating B lymphocytes change their mitochondrial metabolism and how this impacts antitumor immune responses 74.

### 2.5 The mitochondria of NK cells

NK cells are innate cytotoxic lymphocytes that induce cell death through direct cytotoxicity and modulate a multicellular protective immune response by secreting cytokines and chemokines 75. Additionally, NK cells can interact with dendritic cells, macrophages, and T cells to elicit an immune response 76. An increase in mitochondrial mass and glycolysis occurs in NK cells upon activation, accompanied by higher basal OXPHOS rates. Furthermore, redistribution and accumulation of mitochondria at NK cell immune synapses formed during interactions with tumor cells have been observed 77.

Mitochondrial changes in tumor-infiltrating NK (ITNK) cells impair NK cellular functions in the TME with increased mitochondrial fragmentation, lower ΔΨm, and increased mtROS 78. Metabolic deficits in peripheral blood NK cells from metastatic breast cancer patients can be rescued by blocking TGF-β 79. Mitochondrial dynamics control the mature NK cell fitness. Circulating human CD56^ dim^CD16^+^NK (NKDim) cells have fused mitochondria and higher toxicity than CD56^Br^. Whereas mitochondrial fission and depolarization in NKDim impaired IFN-γ production after IL-2/12 stimulation, resulting in non-specific toxicity, this can be rescued by inhibiting mitochondrial division 80. However, Terren *et al.* showed that cytokine stimulation does not significantly depolarize CD56bright and CD56dim NK cell subsets. IL-12/15/18 stimulation reduces mitochondrial cristae density, possibly through decreased OPA1 expression. Failure to upregulate mitophagy upon stimulation could lead to the accumulation of defective mitochondria, excessive ROS, and cell death. Altered mitochondrial morphology caused by hypoxia promotes tumor immune escape, proliferation, and metastasis 81. NK cells can induce mitochondrial apoptosis in cancer cells, affecting their sensitivity to NK-mediated killing. Preactivated NK cells are resistant to BH3 mimetics, but combining these agents with NK cells can effectively kill cancer cells 82.

### 2.6 The mitochondria of TANs

Neutrophils, derived from bone marrow granulocyte-monocyte progenitor, exhibit anti-tumor activity by antibody-dependent cellular cytotoxicity, direct cytotoxic, and activation of adaptive immunity 83. TANs display both anti-tumor (TAN1) and pro-tumor (TAN2) phenotypes within TME, similar to TAMs 84, 85.

Conventionally, mature neutrophils predominantly rely on glycolysis for energy, although mitochondria regulate apoptosis and neutrophil extracellular traps (NETs) formation. Neutrophils regulate mitochondrial function through glycolysis to produce more mtROS in hypoxia and support the production of pro-inflammatory NETs 86, 87. Neutrophil elastase released from NETs activates TLR-4 on cancer cells, upregulates PGC-1α expression, increases mitochondrial biogenesis, mitochondrial density, ATP production, and oxygen consumption rate, but reduces ΔΨm, thereby promoting tumor growth through metabolic reprogramming of cancer cells 88. This process also promotes tumor growth and metastasis by creating a chronic inflammatory environment. Resting neutrophils have elongated, tubular mitochondria that become fragmented and translocate upon stimulation 89. TANs have more mitochondria and use them to support immune suppression and tumor growth by maintaining NADPH levels and producing ROS 90. Both tumor cells and neutrophils increase glucose uptake, leading to higher lactate levels, which contribute to immunosuppression in the TME.

## 3. Pharmacological Therapies for Regulating Mitochondrial Function in the Treatment of Cancer

Mitochondria play a crucial role in regulating cell metabolism, redox balance, signaling, cell survival, and death of eukaryotic cells 89. Numerous therapeutic approaches have been devised to regulate mitochondrial biological function for precise tumor treatment and recovery of compromised anti-tumor immune responses, ultimately benefiting the prognosis of cancer patients 60, 91. We summarized the anti-tumor pharmacological drugs that regulate the biological function of mitochondria within TME from three aspects: mitochondrial balance (Table 1), energy metabolism, and mtDNA.

### 3.1 Drugs regulating mitochondrial homeostasis

#### 3.1.1 Mitochondrial Dynamics

Mitochondria exhibit morphological heterogeneity through a dynamic equilibrium established between their fusion and fission processes. Alterations in mitochondrial morphology serve as critical indicators of distinct functional states. Mitochondrial fusion is a highly regulated process, mediated by Mitofusin 1 and 2 (MFN1/2) located on the outer mitochondrial membrane, as well as OPA1 situated on the inner mitochondrial membrane 92. Upon upregulation of OPA1, mitochondrial cristae undergo remodeling, becoming denser. This morphological change is accompanied by an increase in OXPHOS activity and a concomitant decrease in glycolysis levels. In contrast, mitochondrial fission, mediated by DRP1 and FIS1, induces an opposing metabolic shift 93.

As mentioned above, mitochondria in CAFs, CSCs, and/or other cancer-associated cells are more fragmented compared with their normal counterparts. Also, the activation process of M1-TAMs, T cells, and NK cells is associated with increased mitochondrial fission, such as T-cell activation mediated by DRP1 60. The selective inhibitor of DRP1, Mitochondrial division inhibitor-1 (Mdivi-1), inhibits proliferation and EMT in thyroid cancer cells by suppressing mitochondrial division 94. Mitochondrial morphology changes to support tumor cell survival or death, highlighting the need for further research on mitochondrial dynamics.

#### 3.1.2 Mitophagy

Mitophagy is a process that removes damaged mitochondria caused by ROS and hypoxia in cancers, promoting aerobic glycolysis and even enhancing drug resistance 95, 96. Receptor-mediated mitophagy, involving proteins such as BCL2-interacting protein 3 (BNIP3), BCL2-interacting protein 3-like (BNIP3L), and FUN14 domain-containing 1, can promote ubiquitin-dependent mitophagy through the PINK1/Parkin pathway 97.

Ketoconazole promoted mitophagy and apoptosis through the accumulation of PINK1 in Hepatocellular carcinoma (HCC) 98. Sirtuin-3, a protein primarily located in the mitochondria that relies on NAD^+^ and promotes mitophagy, was activated by compound 33c (ADTL-SA1215), thereby inhibiting the growth and migration of human breast cancer cells 99. Flubendazole increased DRP1 expression, leading to PINK1/Parkin-mediated mitophagy 100. On the contrary, inhibiting mitophagy may be effective in cancer treatment. THZ-P1-2 induced DNA damage and impaired autophagic flux in leukemic stem cells 101. Treatment with Nitazoxanide induced mtROS production in bladder cancer cells, thereby triggering mitophagy initiation and concurrently inhibiting lysosomal degradation activity. This dual effect subsequently led to impaired mitophagic flux, resulting in the accumulation of damaged mitochondria. Consequently, mitochondrial ROS overload activated apoptosis signaling molecules 102. Therefore, induction or inhibition of mitophagy, a mitochondrial quality control mechanism, is widely regarded as a promising anticancer therapeutic approach.

#### 3.1.3 Apoptosis

Apoptosis, or programmed cell death (PCD), is an 'active death' behavior of cells mediated by gene regulation to maintain the steady state of the intracellular environment 103. Induction of mitochondrial apoptosis has been exploited in clinical oncology to trigger cancer cell death. Myeloid cell leukemia-1 (MCL-1) specific inhibitor A-1210477 efficiently blocks the protective cross-talk between CAFs and cancer cells. Combination treatment of ABT-737 with A-1210477 significantly increases the cellular mortality of both cancer cells and CAFs 104. Piperine (PIP) 105 and Cisplatin 106, 107, and Ethyl acetate extract from Celastrus orbiculatus (COE) 108 caused gastric cancer apoptotic cell death via the mtROS-associated signaling pathway. Trifluoperazine, Mitoxantrone, and Pyrvinium pamoate are mitochondrial inhibitors approved by the FDA and can serve as adjuvant therapies for Temozolomide-resistant Glioblastoma Multiforme (GBM) by inducing mitochondrial intrinsic apoptosis 109. Cinnamaldehyde could not only induce apoptosis in MDSCs but also cause apoptosis in prostate-associated fibroblasts via the intrinsic pathway 110. Wang *et al.* 111 designed and synthesized a novel mitochondrial-targeting compound, HYL001, consisting of Lonidamine (LND), lipophilic triphenylphosphonium (TPP^+^), and an aromatic ring, which serves as the hinge. HYL001 disrupts mitochondria morphology via decreasing the MMP to induce the apoptosis of BCSCs and hepatocellular carcinoma stem cells (HCSCs) by blocking Gln metabolism to amplify mitochondrial oxidative stress.

#### 3.1.4 Redox balance

Maintaining mitochondrial redox balance between ROS and antioxidants is crucial for physiological and pathological processes, and the regulation of this balance can also be used to treat cancer 112. Compared with normal cells, the mitochondria of tumor cells maintain a higher ROS level, causing genomic instability and metastasis 113.

The mitochondria-targeted antioxidant SkQ1 inhibited tumorigenesis in p53 knockout mice at an early stage. Additionally, SkQ1 reverses EMT by inhibiting the expression of mesenchymal N-cadherin 114. Plant-derived polyphenolic compounds are known to act as antioxidants, including Extracts from Olea europaea leaves 115, Sesamol 116, TPP-modified Demethoxycurcumin 117. Apoptosis, DNA fragmentation, and mitochondrial dysfunction are all effects of these compounds, which exhibit strong anticancer activity. The natural flavonoid Baicalein 118, which contains a phenolic hydroxyl group, produces hydroxyl radicals directly through a Fenton-like reaction with copper in cells, generating ROS and leading to genomic DNA damage 119. Furthermore, the presence of excessive ROS in the TME, potentially generated by CAFs or stromal cells 120, can trigger the activation of immunosuppressive cells such as T cell exhaustion, macrophage M2 polarization, and neutrophil formation of NETs. Targeting these cells to diminish their ROS production may be a promising approach for therapeutic intervention. MtROS play significant roles in immune signaling pathways affecting T cell activation, proliferation, and apoptosis, as well as B cell activation 121. Alleviating T cell exhaustion and retaining responsiveness to checkpoint blockade can be achieved by mitigating ROS or hypoxia 71. Inducing ROS and blocking antioxidants may produce side effects in normal cells, so these agents must be more specific and efficient against tumor cells.

#### 3.1.5 Biogenesis

Biogenesis of mitochondria is a dynamic process of continuous mitochondrial production that maintains the structure, number, and function of mitochondria in response to stress 52. Nuclear transcriptional coactivator PGC-1α is essential for mitochondrial biogenesis and binds to a variety of nuclear receptors, including PPARs, the estrogen-related receptor (ERRα), and NRF1/NRF2 122. Increasing PGC-1α activates transcription factors, promotes mitochondrial gene expression, and enhances OXPHOS 123. PGC1α has varying effects in different cancers, with lower levels associated with reduced survival rates for breast cancer 124, HCC 125, and osteosarcoma 126. Boosting mitochondrial biogenesis through PGC1α may enhance cancer cell apoptosis. However, PGC-1α-mediated FAO and OXPHOS contribute to stem cell generation in hepatocellular carcinoma 127. XCT790 128-130 and SLU-PP-1072 131, which are selective ERRα/γ inverse agonists, enhance chemosensitivity and suppress mitochondrial biogenesis in cancer cells. Fenofibrate 132 increases mitochondrial fatty acid catabolism, OXPHOS, and glycolysis to boost the proliferation of TN and CD8^+^ T cells. The mammalian target of rapamycin (mTOR) pathway, regulated by the PI3K-PKB (AKT)-mTOR, supports TN differentiation to TE. PI3K inhibitor Idelalisib 133 keeps CAR-T cells less differentiated, resulting in prolonged survival and improved antitumor abilities. Chemotherapy combined with mitophagy inhibitors (e.g., Mdivi-1 and 188Re-Liposome) sensitizes tumor cells to apoptosis and inhibits metastasis, resulting in tumor eradication 48.

#### 3.1.6 Mitochondrial protein synthesis and degradation

Mitochondria contain over 1,000 proteins, with most being encoded by nuclear DNA. However, 13 key proteins are encoded by mitochondrial DNA and translated within the mitochondria. Several cancer cell lines have shown that Tigecycline reduces mitochondrial ATP production, resulting in slowed cell proliferation 134. Mitochondria have a distinctive protein hydrolysis system involving more than 45 proteases, including respiratory complexes subunits and translocases, to maintain mitochondrial protein homeostasis by eliminating impaired and misfolded proteins 135.

Caseinolytic protease P (ClpP) is upregulated in human tumors, supporting proliferation and desensitizing cells to cisplatin 136. Phenyl ester compounds (AV167, TG42, and TG43) inhibit ClpP activity and induce apoptosis in hepatocyte-derived carcinoma cells 137. Conversely, ZK53, a selective activator of mitochondrial ClpP, inhibits lung squamous cell carcinoma by inhibiting OXPHOS and degrading ETC subunits 138. ONC201 and its more potent analog ONC212, induce cancer cell death by suppressing dopamine receptor D2 and promoting activation of ClpP, leading to decreased ETC activity and increased ROS production 139. ONC201 also switches macrophages to a pro-inflammatory state and inhibits OXPHOS in macrophages, enhancing the glycolytic ATP production and altering glutamate transport 140. Spermidine (SPD) can enhance mitochondrial respiratory function in CD8^+^ T cells and potentially improve cancer immunotherapy 141. Thus, mitochondrial proteins could be a potential target for treatment.

#### 3.1.7 Mitochondrial transfer

Mitochondrial transfer can not only maintain metabolic homeostasis in the tissue microenvironment but also ensure intercellular communication under physiological conditions, whereas it can be impaired by cancer or other diseases. Researchers have described cancer cells absorbing mitochondria from non-malignant TME cells as a novel way to enhance tumor activity and immune escape 142. Mitochondria transfer takes place between cells mediated by intercellular nanotubes, macrovesicles, cell junctions, and fusion 143. Mitochondria transportation potentially contributes to cancer cell migration via reprogramming the metabolism of cancer cells.

Human breast cancer cells (MDA-MB-231) that received mitochondria from NK cells and CD3^+^/CD8^+^ T cells showed higher basal and spare respiratory capacities and lower metabolic mitogenic capacities. An aggressive immunocompetent breast cancer model treated with L-778123 and α-PD1 exhibited reduced nanotube formation and mitochondrial transfer, as well as improved antitumor activity 14. Mitochondrial transfer from neural cells emerges as a key mechanism linking neural density to cancer progression. Astrocytes donate mitochondria to glioblastoma, fueling tumor growth via enhanced respiration and metabolic reprogramming 144. Similarly, breast cancer cells (e.g., 4T1) acquire neuronal mitochondria via TNTs, augmenting OXPHOS, stemness, and stress resistance to drive metastasis 145. These findings define the mechanistic basis of the association between neural density and poor prognosis, establishing a rationale for therapies targeting nerve-cancer metabolic crosstalk, such as local nerve blockade.

### 3.2 Drugs targeting mitochondrial energy metabolism

#### 3.2.1 TCA cycle

The TCA cycle is a central hub that regulates cellular energy metabolism in the mitochondrial matrix. It converts metabolites of glucose, fatty acids, and amino acids into carbon dioxide, generating NADH and FADH2 as electron donors for ATP production through OXPHOS. Mutations in TCA enzymes (e.g., SDH, isocitrate dehydrogenase (IDH), and fumarate hydrogenase (FH)) have been reported in a variety of cancers. SDH and FH mutations lead to the accumulation of succinate and fumarate, respectively, and induce a glycolytic shift, causing a shift to glycolysis 186. In contrast, IDH mutations convert α-ketoglutarate (α-KG) to 2-hydroxyglutarate (2-HG), a byproduct that inhibits α-KG-dependent enzymes, contributing to tumorigenesis. Devimistat (CPI-613), a lipoic acid analog, inhibits pyruvate dehydrogenase (PHD) and α-ketoglutarate dehydrogenase (α-KGDH) 187. Lactate levels gradually rise over time and shift from promoting T cells' function to disrupting them in the tumor microenvironment. Dichloroacetate (DCA) specifically activates PDH, thereby promoting oxidative metabolism and glucose carbon flux through the TCA cycle. Tumors undergoing DCA treatment have their metabolism changed from glycolysis to OXPHOS, significantly increasing T cell function and cytokine production and rescuing them from apoptosis 188.

#### 3.2.2 OXPHOS

The higher energy demand by the cancer cell is achieved by adjusting its metabolism, relying on aerobic glycolysis for energy instead of OXPHOS, as normal cells do. Biguanide antidiabetic drugs and IACS-010759, as mitochondrial complex I inhibitors, can be used for cancer treatment; however, their efficacy is limited due to the activation of metabolic compensation 19. However, inhibition of OXPHOS induces mitochondria to transfer from bone marrow MSCs to AML cells via TNTs; the inhibitor also induces mitochondria division and mitophagy in AML cells, thereby promoting cancer cell migration 20. The activity of mitochondrial complex I in the mitochondrial ETC decreases after Fenofibrate treatment, inhibiting gastric cancer cells' growth *in vivo* through reversing metabolic reprogramming and inducing apoptosis 189. The Mito-FFa immunogenic death inducer, synthesized by TPP^+^ and Fenofibric Acid (FFa), targets mitochondria to increase the mtROS level of tumor cells, also inducing CD8^+^ T cell-mediated antitumor immunity 190.

New studies validate novel immunomodulatory effects of TPP^+^-dependent mitochondria-targeted drugs 191. Mito-magnolol (Mito-MGN), a mitochondria-targeted derivative of magnolol, inhibits melanoma cell proliferation and OXPHOS more effectively than untargeted magnolol 192. Mito-MGN, along with others like Mito-honokiol and Mito-metformin, inhibited pro-tumorigenic factors in the tumor immune microenvironment (TIME). Hydroxyurea (HU) modified with TPP^+^ inhibited more mitochondrial oxygen consumption than HU, potently inhibited tumor cell proliferation, M-MDSCs differentiation, and suppressor neutrophil activation, and stimulated TE response 193.

#### 3.2.3 FAO

Cancers such as lymphomas and leukemias require elevated FAO for ATP production to survive 194. Overexpression of Carnitine palmitoyltransferase-1 (CPT-1) is linked to cancer progression in breast, prostate, and lymphoma cancers 195. The CPT-1 inhibitor Etomoxir has been shown to decrease proliferation in TNBC 196. However, Etomoxir has been discontinued from clinical use due to toxicity at high doses. Melanomas drive FAO in DCs by upregulating CPT1A fatty acid transporter, inducing Treg generation. Etomoxir blocks this pathway, enhancing anti-PD-1 antibody immunotherapy activity 197. A selective and reversible CPT-1A inhibitor, also known as Teglicar, has been demonstrated to repress FAO in leukemia cells, leading to mitochondrial damage as well as apoptosis 198. An inhibitor of CPT-1 with a higher specificity, perhexiline, which inhibits both CPT1 and CPT2, has been investigated in mouse studies for numerous cancer types, such as colorectal cancer cells 199.

#### 3.2.4 Glutamine metabolism

Reprogramming glutamine metabolism is crucial for tumor and immune cell survival in the TME. Tumor cells and immune cells compete for glutamine uptake, limiting its availability for T lymphocytes and impacting anti-tumor immune responses in TNBC. By converting glutamine into glutamate via glutaminase (GLS), glutamate is further converted by glutamate dehydrogenase (GDH) into α-KG, which can be used as an intermediate in the TCA cycle. Human malignancies suffer from high levels of GLS1, including breast, prostate, esophageal, and liver cancers 200. Thus, inhibitors of GLS, such as BPTES and CB-839, demonstrate remarkable antiproliferative activity in tumor cells 201. The anabolic pathway of glutamine is upregulated in CAFs compared to NOFs, providing nutrients for cancer cell growth. Glutamine metabolism induces the differentiation of M2-TAMs, and its inhibition may cause tumor shrinkage. On the contrary, inhibition of glutamine metabolism in NK cells is accompanied by suppression of their anti-tumor function 202.

### 3.3 mtDNA damage

The mitochondrial genome is capable of encoding 13 ETC subunits, mitochondrial rRNAs, and tRNAs, independently of nuclear genes. MtDNA has a high mutation rate and affects tumorigenesis 203. Decreased levels of the mtDNA common deletion have been found in certain types of skin cancer and CAFs, which correlate with increased expression of mtDNA-encoded genes and improved mitochondrial function 204. Mutations in ATP synthase gene reduce apoptosis and promote tumor growth. Mutations in mitochondrial Complex I promote ROS production, leading to metastasis of Lewis lung cancer mouse cells. Cancer cells may accumulate abnormal amounts of mtDNA in the cytoplasm as a result of oxidative stress and mitochondrial dysfunction 205. MtDNA exhibits a dual function in modulating the TME; while mutations in mtDNA facilitate cancer progression and resistance to therapy 55, damaged mtDNA taken up by DCs stimulates IFN-I response and improves antigen presentation. Additionally, radiotherapy-induced damaged mtDNA enhances the anti-tumor function of CD8^+^ T cells 206. Certain chemotherapeutic agents (cisplatin, paclitaxel), metal ions (Mn²⁺, Zn²⁺, Ca²⁺), and ROS act as pyroptosis inducers in tumor cells. This process concurrently releases damaged mtDNA, which activates the cGAS-STING pathway to enhance immunity 207. Yu *et al.* employed cobalt fluoride (CoF₂) nanozymes to induce pyroptosis under ultrasound, generating ROS and damaged mtDNA that activate cGAS-STING 208. Alternatively, gas therapy combined with Mn²⁺ induces significant mitochondrial dysfunction, thereby impeding cancer proliferation 209.

Shen *et al.* 210 found significantly reduced mtDNA content in most Pediatric high-grade gliomas (pHGGs) compared with normal brain, resulting in the metabolic transition toward aerobic glycolysis. They adopted a combination therapy in which dichloroacetate and metformin synergized with radiotherapy (RT), leading to a reversal of the metabolic phenotype, primarily by inducting oxidative stress. DCA is used to shift glucose metabolism toward mitochondrial oxidation. In contrast, metformin targets mitochondrial function while disrupting the tumor's energy balance, thereby increasing DNA damage and apoptosis. OXPHOS-dependent CSCs are targeted with first-in-class, specific mitochondrial transcription inhibitors (IMTs), targeting human mitochondrial RNA polymerase (POLRMT), as an enzyme that plays a crucial role in its biogenesis 211.

## 4. Nanotechnology-Based Therapeutics for Regulating the Mitochondria of Tumors or Tumor-Associated Cells

As previously discussed, despite substantial research on pharmacological agents to modulate mitochondrial function in tumor and tumor-associated cells, precise mitochondrial drug targeting remains challenging due to the dual barriers of the mitochondrial double-membrane structure and TME complexity 212. The rapid advancement of nanotechnology continues to revolutionize conventional pharmacology. NDDS confer multiple advantages, including enhanced stability, superior targeting capability, and improved bioavailability for highly potent therapeutic agents 213, 214. Recent years have witnessed remarkable progress in mitochondria-targeted nanotherapeutics designed for both malignant and stromal cells. These tailored nanoplatforms enable precise mitochondrial accumulation and potentiated regulation of mitochondrial functions. More significantly, synergistic integration of mitochondria-targeted nanodrugs with chemotherapy, PDT, SDT, RDT, magnetic hyperthermia therapy (MHT), photothermal therapy (PTT), and immunotherapy has demonstrated enhanced efficacy in combating tumor progression 24, 215 (Table 3).

### 4.1 Nanodrugs regulating mitochondria of tumor cells and immunogenic cell death effect

**Figure 5:**
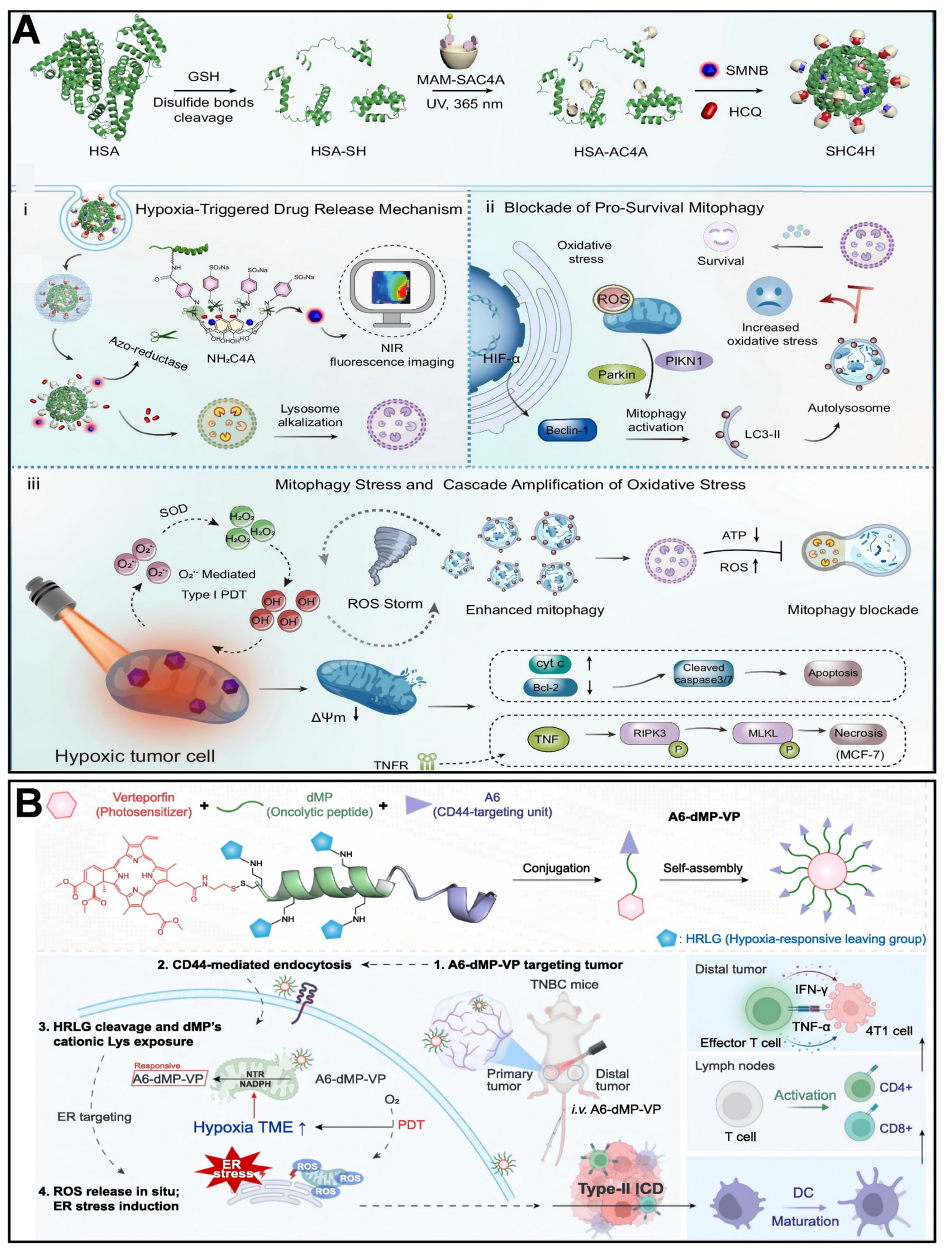
(A) Design and hypoxia-activated antitumor action of SHC4H. (B) Schematic design and mechanism of action of the hypoxia-responsive lysosomal coupling A6-dMP-VP. (A) Reproduced with permission from 216, copyright 2025, Springer Nature Limited. (B) Reproduced with permission from 27, copyright 2025, Elsevier B.V.

**Figure 6:**
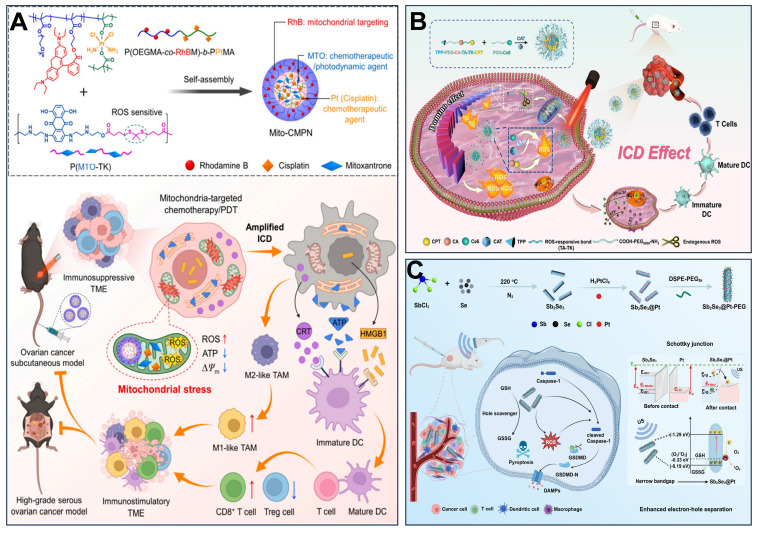
(A) Mito-CMPNs-mediated mitochondria-targeted photodynamic therapy (PDT)/chemotherapy and its unique ability to improve cancer treatment in terms of immunostimulation. (B) Schematic illustration of ROS-responsive, mitochondria-targeted micelles for enhanced ICD via a domino effect of amplified oxidative stress. (C) Diagram showing the synthesis of Sb2Se3@Pt with a Schottky junction, and the mechanism of SCT therapy in modifying the tumor microenvironment to boost pyroptosis-mediated immune response against cancer. (A) Reproduced with permission from 217, copyright 2024, Elsevier B.V. (B) Reproduced with permission from 218, copyright 2024, Elsevier B.V. (C) Reproduced with permission from 221, copyright 2024, Wiley-VCH GmbH.

Wang *et al.* 216 developed a novel SHC4H nanoparticle, which combines chemotherapy and mitochondria-targeting photodynamic therapy (M-PDT). Azocalix 4 arene (AC4A) was modified onto human serum albumin (HSA) to form the HSA-AC4A carrier, which was co-loaded with hydroxychloroquine (HCQ) and a mitochondria-targeting Type I photosensitizer (SMNB). Utilizing the hypoxia-responsive property of AC4A, azobenzene bonds were reductively cleaved in the hypoxic tumor microenvironment, thereby releasing HCQ and SMNB. HCQ blocked mitophagic flux by alkalinizing lysosomes, while SMNB generated ROS via an electron transfer mechanism under 660 nm laser irradiation, damaging mitochondria and activating mitophagy. In hypoxic tumor cells, the dual attack of HCQ-induced mitophagic flux blockade and SMNB-triggered mitochondrial damage was achieved (Figure 5A). Growing evidence suggests that inducing mitochondrial stress is often more effective than triggering large-scale induction of ICD via endoplasmic reticulum (ER) stress. Ren *et al.* 27 designed and synthesized a tumor-targeting and hypoxia-responsive peptide-photosensitizer coupling, A6-dMP-VP, which consists of a CD44 targeting motif, A6, a tumor lysing peptide, dMP, a hypoxia-responsive motif, and a photosensitizer, vitexoporfin (VP), which self-assembles into nanoparticles and is preferentially enriched in 4T1 tumors. In the hypoxic tumor microenvironment, mitochondrial nitroreductase (NTR) cleaves the hypoxia-responsive motifs. It releases the cationic dMP, which binds to and destroys the mitochondrial membrane through electrostatic interaction. At the same time, VP generates ROS by light exposure, which exacerbates endoplasmic reticulum stress, and the two synergistically induce type II immunogenic cell death (Figure 5B). Zhang *et al.* 217 designed mitochondria-targeted multi-premedicine nanoparticles, Mito-CMPN, which target mitochondria via rhodamine B (RhB) and piggyback on cisplatin and mitoxantrone (MTO). Under laser irradiation, MTO generates ROS to trigger photodynamic therapy, while the nanoparticles release drugs in response to ROS and GSH in the tumor microenvironment, synergistically inducing mitochondrial stress. This process prompts tumor cells to release immunogenic molecules (e.g., CRT, HMGB1, ATP), activates dendritic cells, polarizes M2-type macrophages to M1-type, and enhances CD8^+^ T-cell infiltration, reversing the immunosuppressive microenvironment (Figure 6A). Hu *et al.* 218 developed ROS-responsive mitochondria-targeted micelles (CTC) co-delivering CPT, Ce6, and CAT to mitochondria via TPP. The triple synergy of CPT-induced ROS, Ce6-mediated photodynamic ROS, and CAT-catalyzed O₂ generation created a sustained ROS cascade (domino effect), leading to mitochondrial membrane collapse, ER stress activation, and immunogenic cell death (Figure 6B). Cheng *et al.* 30 developed a semiconductor polymer nanomessenger (TCa/SPN/a) co-loaded with Ca²⁺ and cGAMP. Under X-ray irradiation, it generated ROS via a radiodynamic effect to induce ICD, while simultaneously releasing mitochondria-targeted Ca²⁺ to trigger calcium overload and mitochondrial damage. This cascade activated the STING pathway, enhancing the immunotherapeutic effect and significantly suppressing tumor growth and metastasis in the breast cancer model.

Furthermore, pyroptosis can also induce ICD. This process is characterized by transmembrane pore formation, cellular swelling, and lysis, culminating in the release of inflammatory factors and intracellular contents. Cheng's team investigated the link between mitochondrial damage and pyroptosis. They developed nanoparticle platforms combining metals or metal oxides with therapies such as MHT, SDT, immunotherapy, transarterial embolization (TAE), and sonocatalytic therapy (SCT) to induce tumor mitochondrial damage, pyroptosis, and antitumor immunity 30, 219-221. Han *et al.* 219 synthesized Zn-LDH@Mg implants by a hydrothermal method, which had rough surfaces with Zn-layered double hydroxides (LDH) on the Mg implants. Zn-LDH@Mg induced Caspase/Gasdermin D (GSDMD)-dependent pyroptosis in hepatoma cells, enhanced by increased Zn²⁺ uptake amplifying mitochondrial oxidative stress. Resultant pyroptosis triggered ICD via “eat me” signals (e.g., calreticulin exposure). Sun *et al.* 30 constructed fluorinated titanium dioxide (TiO₂-ₓFₓ) sonosensitizers. Under ultrasound, these nanoparticles generated excessive ROS that induced mitochondrial damage and oxidative homeostasis disruption, ultimately triggering significant pyroptosis. Notably, SDT-facilitated pyroptosis prevented tumor recurrence by initiating a potent immune memory effect. Wang *et al.* 220 designed an innovative magnetic metallo-immunotherapeutic platform based on Zn-Fe₃O₄@Co-Fe₃O₄ core-shell nanocubes (ZnCo-Fe₃O₄ CSNCs). This platform integrates TAE, MHT, and immunotherapy. Under an alternating magnetic field (AMF), temperature-dependent release of Fe²⁺ and Zn²⁺ ions disrupted mitochondrial morphology, causing extensive vacuolation, membrane potential depolarization, and reduced ATP levels. Crucially, by suppressing ATP synthesis, this process also lowered heat shock protein (HSP) expression, thereby inducing GSDMD-dependent pyroptosis in hepatocellular carcinoma cells and subsequent immune activation. Nie *et al.* 221 developed a sonocatalytic platform, Sb_2_Se_3_@Pt core-shell heterostructures, that initiates pyroptosis to enhance SDT-immunotherapy. Under ultrasound (US) irradiation, Sb_2_Se_3_@Pt generates excessive ROS, inducing mitochondrial dysfunction and pyroptosis (Figure 6C). Guo *et al.* 222 prepared VB12-sericin-PBLG-IR780 nanomicelles to induce pyroptosis and stimulate antitumor immunity. Combined PTT and PDT, ATP synthase expression could be inhibited, and mitochondria were damaged by nanomicelles, leading to mtROS-induced mtDNA damage and pyroptosis through NLRP3/Caspase-1/gasdermin D pathway and DCs maturation. These studies demonstrate that mitochondrial dysfunction accompanies, and may even promote, the induction of tumor pyroptosis.

### 4.2 Nanodrugs regulating mitochondria of tumor-associated myeloid cells (TAMCs)

**Figure 7:**
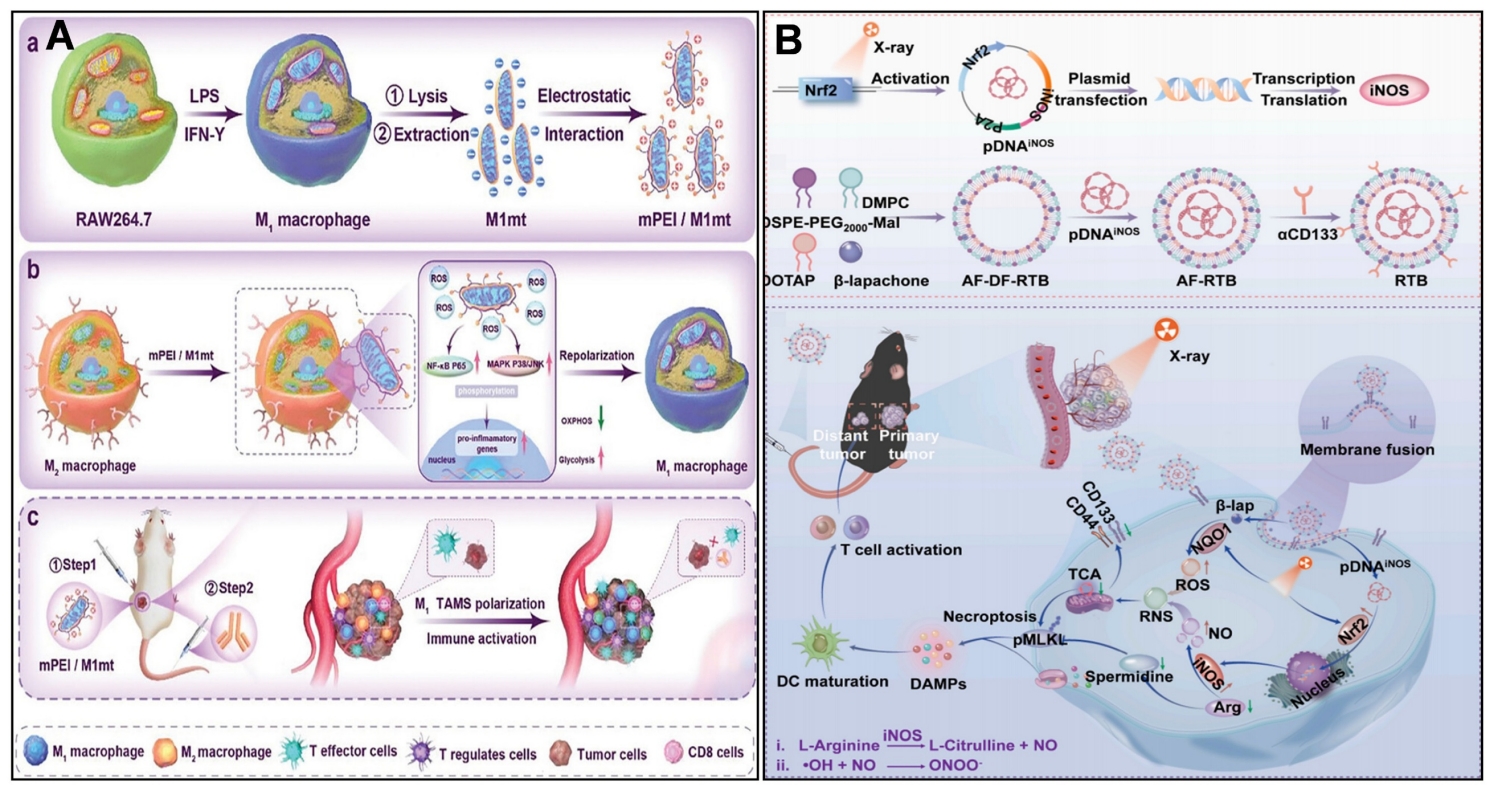
(A) Schematic representation of engineered mitochondria reprogramming M2 TAM to produce inflammatory TME and enhance cancer. (B) Illustration of RTB synthesis and its mechanism for eliminating CSCs by enhancing radio-immunotherapy. (A) Reproduced with permission from 223, copyright 2024, Wiley-VCH GmbH. (B) Reproduced with permission from 227, copyright 2025, Elsevier B.V.

TAMCs are heterogeneous cells that infiltrate tumors and influence tumor growth profoundly, including TAMs, DCs, and neutrophils. Targeting mitochondria in TAMs reprogrammed TAMs from pro-tumor to anti-tumor, as a potential therapeutic strategy. Zhang *et al.* 223 designed a mannose-modified mitochondria-targeted delivery system (mPEI/M1mt), in which M1-type macrophage-derived mitochondria were electrostatically encapsulated in mannose-modified polyethyleneimine, and targeted uptake was achieved by utilizing the mannose receptor, which is highly expressed on the surface of M2-TAMs. mPEI/M1mt promoted M2-TAMs' metabolism through induction of a shift in the M2-TAMs' metabolic By inducing the metabolic pattern of M2-TAMs to shift from OXPHOS to glycolysis, mPEI/M1mt elevates the intracellular ROS level and activates the NF-κB p65, MAPK p38, and JNK signaling pathways, which in turn promote the polarization of M2-TAMs toward the pro-inflammatory and anti-tumor M1 phenotype (Figure 7A).

Sharma *et al.* 224 presented a dendrimer-mediated nanomedicine (D-DPA) that targets TAMs in glioblastoma. Generation four hydroxyl-terminated polyamidoamine (PAMAM) dendrimer was clicked by 5,7-dimethylpyrazolo[1,5-α] pyrimidin-3-ylacetamide (DPA), which can specifically target translocator protein (TPSO). D-DPA targeted specifically to immunosuppressed TAMs due to high TPSO expression. D-DPA showed stronger TPSO affinity than the free TPSO ligand PK_11195_, and was more co-located with mitochondria than the unmodified dendrimers. D-DPA upregulates the antitumor immune signaling of TNF-α and IL-1β, as well as inhibits the expression of protumor signal of Arg-1. Zhao *et al.* 225, 226 used P/shMFN1 NPs to repolarize pro-tumor TAMs to an anti-tumor phenotype by down-regulating the expression of the mitochondrial fusion gene. Mitofusin 1 shRNA (shMFN1) was loaded with Man-PEI-PCL, then coated with 2, 3-dimethylmaleic anhydride modified polyethylene glycol shell (PEG-DMMA) to form P/shMFN1. P/shMFN1 targeted M2-TAMs with mannose guidance to shMFN1, resulting in mitochondrial fragmentation and increased ROS generation. Consequently, M2-TAMs (F4/80^+^CD206^+^) shifted to M1-TAMs (F4/80^+^CD80^+^) through mitochondrial fission. They also designed another nanoparticle, P-aCD24/CEL, to target tumor cells, inducing ICD of tumor cells and reactivating the immune regulation of macrophages. Enhancing antitumor immune responses by targeting tumor cells and TAMs, respectively.

### 4.3 Nanodrugs regulating mitochondria of CAFs

Tumor stroma is dominated by CAFs, which secrete pro-tumor growth factors that enhance solid tumor pressure and interstitial fluid pressure (IFP). Targeting receptors overexpressed on CAFs can improve the effectiveness of nanotherapy, including fibroblast activation protein (FAP), sigma receptor, angiotensin II type I receptor, and tenascin C protein. Chemotherapy-induced ROS stimulates CAFs that confer chemoresistance via mitochondrial transfer through TNTs. Tumor-derived vesicles additionally activate CAF mitophagy, boosting mtDNA release, which drives metastasis in therapy-damaged lung cancer cells 228.

Li *et al.* 229 developed a diselenium-bonded organosilicon nanodelivery system (Se@A&F) targeting FAP, which specifically acts on CAFs in prostate cancer by carrying CXCR4 antagonist AMD3100. Se@A&F induced CAFs' inactivation and remodeled the tumor microenvironment by down-regulating CXCR4 expression on CAFs' surface and inhibiting the CXCL12/CXCR4 signaling axis. Se@A&F induces CAFs' inactivation and remodels the tumor microenvironment by down-regulating CXCR4 expression on CAFs' surface and inhibiting the CXCL12/CXCR4 signaling axis. Meanwhile, selenium released from the nanosystem induces mitochondrial dysfunction in tumor cells, triggering apoptosis of RM-1 tumor cells by generating ROS and disrupting MMP. Sitia *et al.* 242 developed a CAF-targeted pro-apoptotic Antibody-Drug Conjugate (ADC). Firstly, Navitoclax (Nav) was coupled with anhydrous CuSO4 (II), incubated with H-ferritin (HFn) to form HNav due to the affinity between metal ions and protein. Then, functionalized HNav-Fab consisted of HNav nanocages with the Fab fragment of an anti-FAP antibody (Fab@FAP) via a heterobifunctional linker NHS-PEG-Mal. Functionalizing HFn with FAP antibody fragments can significantly enhance the CAF-specific delivery of the drug and compete with the TfR1 receptor, thereby decreasing off-target distribution in tumor cells. The results showed that HNav-FAP targeted and induced apoptotic cell death by activation of the mitochondria pathway in FAP+ breast cancer cells. Therefore, HNav-FAP, when combined with chemotherapeutics, could be a promising therapeutic strategy to reduce the formation of metastases.

Mitochondrial double-stranded RNA (mt-dsRNA) from B-cell precursor acute lymphoblastic leukemia (B-ALL) cells induces CAF differentiation from MSCs through interferon activation. Resulting CAFs support chemoresistance by mitochondrial transfer via TNTs. Disrupting dsRNA prevents CAF generation, offering therapeutic potential 230.

### 4.4 Nanodrugs regulating mitochondria of CSCs

CSCs are implicated in tumorigenesis, recurrence, metastasis, and drug resistance. Of particular significance is the pivotal role of mitochondria in CSCs, prompting the exploration of anti-tumor stem cell therapeutic approaches that target mitochondria. Several aspects of mitochondrial morphology and structure, subcellular localization, mtDNA, metabolic status, and mitophagy activity determine the function and fate of CSCs.

CSCs depend on mitochondrial OXPHOS to maintain their stemness and therapy resistance, and arginine metabolism plays a key role in this process. Yao *et al.* 227 investigated a nano-integrated radiation-triggered bioreactor (RTB). This RTB system depletes arginine within CSC through radiation-induced iNOS expression, while generating NO to enhance the cellular toxicity and disrupt mitochondrial function in concert with β-lapachone (LAP)-generated ROS. This dual action drives the transition of CSC to a differentiated state and induces immunogenic cell death through the activation of RIPK1-dependent necrotic apoptosis, thereby stimulating a durable anti-tumor immune response (Figure 7B). Xiao *et al.* 231 investigated and developed the ROS-responsive targeting nanodrug CuET@PHF, which stabilizes the copper ion carrier CuET nanocrystals by dopamine and hydroxyethyl starch, and utilizes folic acid for active targeting. The drug induces copper death in cancer cells and CSCs by targeted delivery of copper ions to mitochondria, thereby disrupting tricarboxylic acid cycle proteins and triggering cell death. Zhang *et al.* 232 designed a bis(diethyldithiocarbamate)-copper/indocyanine green nanoparticles (CuET/ICG NPs) prepared by one-pot method, which could alleviate tumor hypoxia by inhibiting mitochondrial oxygen depletion and enhance PDT-induced immunogenic cell death, and at the same time activate the AMPK pathway by triggering energetic stress through mitochondrial dysfunction to down-regulate PD-L1 expression on the surface of CSCs and tumor activate AMPK pathway through mitochondrial dysfunction, down-regulate PD-L1 expression on the surface of CSCs and tumor cells, and relieve immunosuppression.

### 4.5 Nanodrugs regulating mitochondria of T cells

**Figure 8:**
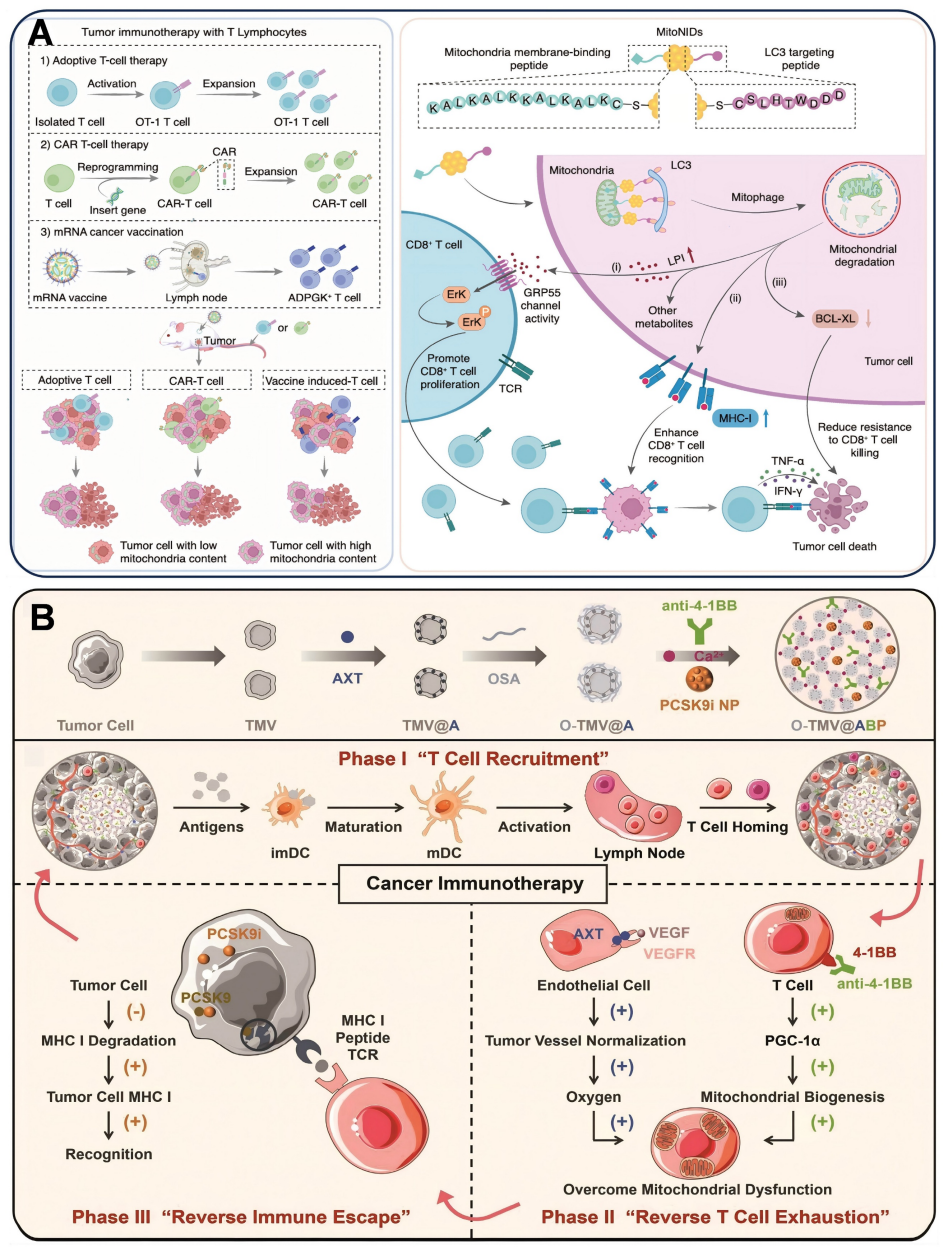
(A) Schematic overview of how mitoNID promotes mitochondrial degradation for optimized CD8^+^ T cell immunotherapy. (B) Schematic illustration of the preparation of O-TMV@ABP injectable hydrogel and its role in T cell recruitment, alleviation of T cell exhaustion, and overcoming MHC I-mediated immune evasion. (A) Reproduced with permission from 233, copyright 2025, Springer Nature Limited. (B) Reproduced with permission from 234, copyright 2022, Wiley-VCH GmbH.

Mitochondrial dysfunction in CD8^+^ T cells leads to T cell exhaustion, which weakens their antitumor effects. Modulation of energy metabolism, redox balance, and dynamics of mitochondria in CD8^+^ T cells can remodel their antitumor functions. Tumor cell mitochondrial content is positively correlated with their resistance to CD8^+^ T cell killing, and tumor cells with high mitochondrial content are more likely to evade attack. mitoNIDs developed by Pan *et al.* were constructed by using gold nanoparticles (GNPs) as carriers, and modifying the mitochondrial membrane-bound peptide (KALKALKKKALKALKC) and LC3-targeting peptide (DDDWTHLSC) by gold-sulfur bonding to create a nano-agent with both mitochondrial targeting and autophagy activation functions. DDDWTHLSC). It not only enhances the recognition and activation of CD8^+^ T cells on tumor cells, but also improves the sensitivity of tumor cells to CD8^+^ T cell killing, which provides a new strategy to enhance the effect of tumor immunotherapy 233 (Figure 8A).

Insufficient major histocompatibility complex class I (MHC I) on tumor cells due to subtilisin/kexin type 9 (PCSK9) degradation hinders T cell recognition, reducing therapeutic effectiveness. Zhang *et al.* 234 designed an injectable hydrogel O-TMV@ABP that encapsulated axitinib (AXT), 4-1BB antibody, and PF-06446846 (PCSK9 inhibitor) in gelator oxidized sodium alginate-modified cancer cell membrane vesicles (O-TMV). The hydrogel worked in three steps. In the first stage, O-TMV triggered an immune response at the tumor site. In the second phase, the 4-1BB antibody and AXT reverse TEX. In the third phase, PF-06446846 enhanced MHC I expression on the tumor and promoted T cell recognition of tumor cells. This injectable hydrogel reprogrammed T cells to recognize tumor cells, thereby facilitating T cell-based cancer immunotherapy (Figure 8B). CAFs create a barrier to prevent TILs from entering the tumor by secreting collagen, FAP, and chemokine. Platelet membrane (Pm)-encapsulated magnetic metal-organic framework nanoplatforms composed of PmMN@Om&As. They simultaneously deliver oxymatrine (Om) and astragaloside IV (As) at the tumor site to inactivate CAFs and augment the level of TILs. The magnetic field and Pm coating help deliver Om and As to HCC cells. Inhibiting CAFs activation with Om can increase TIL levels and enhance mitochondrial function by upregulating PGC-1α. Combining PmMN@Om&As with α-PD-1 improves anti-HCC effects, prolonging survival. Enhancing TIL activity with As is an effective strategy 235. Chen *et al.* 236 developed a liposomal nanoreactor (L@Mn@SPD) that forms Mn(OH)₂ precipitates within liposomes, co-delivering Mn²⁺, SPD, and O₂ to the TME. This nanoreactor alleviates TME hypoxia, simultaneously activating Mn-based STING immunostimulation and countering T cell exhaustion by enhancing mitochondrial respiration and ATP production. This approach overcomes the limitation of T cell mitochondrial dysfunction that compromises Mn-based cancer immunotherapy.

## 5. Discussion

Our analysis compares pharmacological agents and nanomedicines targeting mitochondria in tumor and tumor-associated cells, revealing fundamentally distinct design paradigms despite a shared therapeutic goal. Small molecules inherently rely on systemic exposure, prioritizing strategies like lipophilic cations (e.g., TPP⁺, DQA) for membrane potential-driven accumulation or substrate analogs (e.g., DCA, CPI-613) for enzymatic affinity to achieve sufficient mitochondrial residence 111, 117, 188. Conversely, nanomedicines actively circumvent systemic exposure (a major source of side effects), leveraging the TME (pH, enzymes, hypoxia, or receptor overexpression) to achieve tumor-specific localization first 244. This enables hierarchical targeting: to first confine the carrier within the tumor region. Subsequently, secondary release of drugs or ions enables hierarchical targeting: achieving "tumor-first" accumulation followed by “mitochondria-second” delivery.

Further distinctions lie in specificity, controllability, scope, and overcoming limitations. Small molecules typically pursue single-target interventions (e.g., DRP1, BCL-2, CPT-1) with fixed pharmacokinetic profiles, limiting spatiotemporal control. Nanomedicines embrace multimodal synergy (e.g., combining chemotherapy, PDT, SDT, PTT, immunotherapy, via mechanisms like ROS cascades, Ca²⁺ overload, ICD, STING pathway activation) and provide dynamic “on-demand” controllability using external/internal triggers (light, US, X-ray, H₂O₂, hypoxia/ROS/pH-sensitive linkers) 71, 215. Critically, while small molecules primarily induce autonomous tumor cell mitochondrial death constrained by chemical draggability (solubility, stability, toxicity), nanomedicines exploit their engineerable nature to overcome these barriers. They deliver “undruggable” payloads (ions like Zn²⁺/Cu²⁺/Ca²⁺, proteins, shRNA, whole mitochondria) and actively reprogram intercellular mitochondrial transfer (e.g., between TAM/CAFs/ T cells and tumor cells), reshaping the immune-stromal network 23. Through material selection (polymers, lipids, metal-organic frameworks, cell membrane coatings) and surface engineering (targeting peptides, antibodies, glycosylation), they transform “undruggable” payloads into “deliverable” therapeutics. In essence, small molecule strategies maximize mitochondrial affinity within the confines of limited chemical space, whereas nanomedicine leverages virtually infinite engineering space to achieve spatiotemporally precise, multi-modal mitochondrial intervention with diverse payloads.

## 6. Conclusion and Future Perspectives

This review summarizes the metabolic reprogramming of tumor-associated cellular mitochondria (TAMs, CAFs, CSCs, T cells, NK cells, TANs) in tumor tissues under the influence of tumor cells. Mitochondria, as the cellular powerhouses, play a key role in adapting to these metabolic changes. Modulation of mitochondrial function in tumor cells and associated cells can effectively impede tumor growth and metastasis, offering novel approaches to combat drug resistance and cancer recurrence. Additionally, targeting the mitochondria of tumors or CAFs, CSCs, TAMCs, and tumor-infiltrating T cells within the TME with nanomedicines can mitigate the immunosuppressive effects and enhance the anti-tumor response, thereby shifting the balance towards anti-tumor immunity.

Despite the significant progress made in current research, therapeutic strategies targeting mitochondria still face many challenges in clinical applications. Future research needs to delve deeper into the specific mechanisms of mitochondrial action in different types of tumors and how to precisely regulate mitochondrial function to optimize therapeutic outcomes. Additionally, the development of novel nanodrug delivery systems to achieve targeted therapy of mitochondria in tumor cells and tumor-associated cells will be an important direction for future research. Combining immunotherapy and metabolic therapy may lead to more significant clinical effects in cancer treatment. Lastly, the advancement of personalized medicine requires a better comprehension of the heterogeneity of the TME and how to tailor mitochondrial-targeted therapeutic strategies according to the specific circumstances of patients. With the continuous progress of science and technology, cancer therapies will increasingly target mitochondria in the future.

The following aspects should be addressed in future research to achieve these goals:

(1) Gain a comprehensive understanding of the mechanisms underlying mitochondrial function in various tumor-associated cells within the TME and their impact on tumor development and treatment responses. Investigate the interplay between mitochondria and other cell types in the TME, and assess how these interactions influence tumor progression and treatment outcomes.

(2) Develop and optimize nanotechnologies to improve the targeting and efficiency of drug delivery, reducing side effects.

(3) Explore the synergistic effects of mitochondria-targeted therapy in combination with other treatment modalities (including PTT, immunotherapy, chemotherapy, and radiotherapy) to optimize therapeutic efficacy.

(4) Validate the safety and effectiveness of mitochondria-targeted therapy through clinical trials and develop individual treatment plans according to the genetic background and tumor characteristics of patients.

## Figures and Tables

**Table 1:** Summary of pharmacological agents targeting mitochondrial homeostasis regulation

Regulating mitochondrial function	Tumor model/Cell model	Pharmacological agents	Mechanism of action	Ref.
Mitochondrial dynamics	Thyroid cancer cells, breast cancer stem cells (BCSCs), and pancreatic CSCs.	Mdivi-1	Selective inhibitor of fission protein DRP1, inducing apoptosis in the tumor.	94 146 147
	Lung carcinoma cells	Drpitor1 Drpitor1a	Inhibited the GTPase activity of DRP1, inducing apoptosis.	148
	Nasopharyngeal carcinoma (NPC)	Resveratrol	An inhibitor of Cyclooxygenase-2 (COX-2)/inhibition of DRP1 activation.	149
	Colon carcinoma	Celecoxib	Nonsteroidal anti-inflammatory drug, an inhibitor of COX-2/inhibition of DRP1 activation.	150
	liver cancer-initiating cells (LCICs)	Furamidine	Inhibited mitochondrial fission.	50
	BCSCs	Capivasertib (AZD5363)	The MFN1 inhibitor induced apoptosis in the tumor.	151
	Lung cancer cells	Baicalein	Induced mitochondrial fission, apoptosis, and autophagy.	152
Mitophagy	Hepatocellular carcinoma (HCC)	Ketoconazole	COX2 inhibitor, pro-mitophagy via PINK1/Parkin pathway, induced apoptosis in HCC.	150
	Lung cancer cells	Dihydroergotamide tartrate	Pro-mitophagy via the PINK1/Parkin pathway induced apoptosis and mitophagy.	153
	Triple negative breast cancer (TNBC)	33c (ADTL-SA1215)	Sirtuin-3 inhibitor inhibits autophagy/mitophagy.	99
	TNBC	Flubendazole	DRP1-mediated mitophagy via targeting EVA1A.	100
	Cervical cancer cells	Sorafenib	Inhibited complexes II and III of the ETC to induce PINK1-Parkin-mediated mitophagy.	154
	Glioma cells	AT101	BH3-mimetic, Pro-mitophagy	155
	Pancreas ductal adenocarcinoma	WJ460	Increased mitophagy and ROS accumulation.	156
	Hepatic Cancer Stem Cells	Mdivi-1	The DRP1 inhibitor inhibited mitophagy.	157
	Breast cancer cells	Liensinine	Blocked autophagosome-lysosome fusion at late stages of autophagy/mitophagy.	158
	Colorectal cancer cells	Analogues of Strigolactones	Autophagy/mitophagy inhibitor, blocking the fusion of autophagosomes and lysosomes.	159
	Cervical cancer cells	Melatonin	Inhibited mitophagy by downregulating Parkin, leading to apoptosis	160
	Leukemic stem cells	THZ-P1-2	Inhibitor of phosphatidylinositol-5-phosphate 4-kinase type 2 protein, impaired autophagic flux.	101
	Bladder cancer cells	Nitazoxanide	Impaired autophagic flux, leading to apoptosis.	161
Apoptosis	Small-cell lung cancer and acute lymphocytic leukemia, CAFs in Cholangiocarcinoma	Navitoclax (ABT-263)	BH-3 mimetics, an inhibitor of BCL-2, induce apoptosis.	162 163
	Chronic lymphocytic leukemia, Cholangiocarcinoma-associated fibroblasts, Colorectal cancer cells	Venetoclax (ABT-199)	Inhibited OXPHOS and TCA cycle independent of BCL-2 inhibition.	164 165 166
	Luminal breast cancers and breast cancer-associated fibroblasts	A-1210477	BH-3 mimetics, an inhibitor of antiapoptotic MCL-1, induce apoptosis.	104
	TNBC	CPSI-1306	Macrophage migration inhibitory factor (MIF) inhibitor, inducing apoptosis.	167
	gastric cancer	PIP	Increased ROS production and decreased ΔΨm, inducing intrinsic apoptosis.	105
	Gastric cancer (GC) Ovarian cancer	Cisplatin	Activated the mitochondrial apoptosis pathway, increasing the intracellular ROS, decreasing the MMP, and regulating the related proteins	106, 107
	gastric cancer (GC)	Ethyl acetate extract of COE	Inhibited Prohibitin (PHB) expression,	108
	glioma stem-like cells (GSCs) Pancreatic ductal adenocarcinoma cells	Trifluoperazine	Dopamine receptor antagonist, decreased OXPHOS, and increased ROS, inducing apoptosis.	109 168
	glioma stem-like cells (GSCs)	mitoxantrone	Induced cleavage of Caspase9/3 and apoptosis.	109
	glioma stem-like cells (GSCs)	Pyrvinium pamoate	Inhibited mitochondrial complex I, inducing apoptotic death.	109
	Prostate-associated fibroblasts, colon adenocarcinoma cell line MDSCs.	Cinnamaldehyde	Enhanced proapoptotic activity through anti-topoisomerase I and II, or inhibiting NF-κB and activating protein 1.	110 169
	BCSCs, hepatocellular carcinoma stem cells (HCSCs).	LND	LND, as a glycolysis inhibitor, induced apoptosis of BSCSs and HCSCs.	111
Redox balance	Lymphoma, hepatocellular xenograft tumors.	SkQ1	Mitochondria-targeted antioxidants reduce ROS, inhibit tumorigenesis, and reverse EMT.	114
	Breast and ovarian cancers	Extracts from Olea europaea leaves	Increased mtROS and induction of apoptosis.	115
	Colorectal carcinoma	Sesamol	Induced the mitochondrial apoptosis pathway through pro-oxidant effect.	170
	Glioma cells	TPP-Demethoxycurcumin (DMC-TPP)	Induced mitochondria-dependent apoptosis by activation of caspase.	117
	Breast cancer cells	Baicalein	Production of ROS through the Fenton-like reaction leads to apoptosis.	171
	glioblastoma CSCs	N-heterocyclic carbene (NHC)-Ir(III)	ROS production and necroptosis-like cell death independent of caspase-dependent signaling.	172
	B16 melanoma-infiltrating T cells	N-acetylcysteine (NAC)	Reduced mtROS in CD8^+^ T cells and enhanced immune responses.	173
	Lymphoma and melanoma-infiltrating T cells	Nicotinamide ribose (NR)	Decreased mtROS in CD8^+^T cell, restoring mitophagy and reversing T cell exhaustion.	174
	Human blood monocytes	nanoshutter-1	The NADPH oxidase-2 (NOX2) inhibitor attenuated macrophage differentiation via suppression of NOX2-mediated ROS production.	175
	Human blood monocytes Breast cancer cell	MnTE-2-PyP^5+^ (BMX-010)	The manganese porphyrin family of redox-active drugs inhibited M2 polarization by scavenging ROS.	176
	Breast cancer cell	Kaempferol	Inhibited neutrophil extracellular traps formation by ROS inhibition, and reduced tumor metastasis.	177
Biogenesis	BCSCs Acute myeloid leukemia Pancreatic cancer (PC)	XCT790	Inversed agonist of ERRα, reduces Oxygen consumption rate (OCR) and inhibits mitochondrial biogenesis.	128 129 130
	Prostate Cancer Cells	SLU-PP-1072	Decreased mitochondrial biogenesis.	131
	LLC infiltrating-CD8^+^ T cells Melanoma mice model-specific infiltrating-CD8^+^ T cells	bezafibrate	PGC-1α agonist, increased expression of ROS and PGC-1α in CD8^+^ T cells, and increased mitochondrial biogenesis. Increased FAO and mitochondrial respiratory capacity.	178 179
	Melanoma mice model-specific CD8+ T cells	fenofibrate	Agonists of PPAR-α, increased CD8^+^ T cell function.	132
	B6 SJL mice lymphoma model	Idelalisib	PI3K inhibitor enhanced the anti-tumor activity of OT-T cells.	180
Protein regulation	Acute myeloid leukemia (AML) chronic myeloid leukemia renal cell carcinoma ovarian cancer	Tigecycline	Inhibition of Mitochondrial protein translation.	181
	Hepatocyte-derived carcinoma cells	Phenyl ester compounds	ClpP inhibitor induced apoptosis.	182
	Lung squamous cell carcinoma	ZK53	ClpP activator inhibited cancer cells through degradation of ETC subunits and impaired OXPHOS.	138
	AML Lymphoma	ONC201, ONC212	ClpP agonist, decreased activity of ETC, and increased ROS.	183
	TAMs in ovarian cancer cell	ONC201	Dopamine receptor D2 antagonist; loss of mitochondrial integrity, switching to pro-inflammatory macrophages.	140
	LLC, colon carcinoma cell infiltrating-CD8^+^ T cells	SPD	Enhanced mitochondrial respiratory function in CD8^+^ T cells and anticancer immunotherapy.	141
Mitochondrial Transfer	mesenchymal stem cells (MSCs) / T cell acute lymphoblastic leukemia (T-ALL) cells CAFs / Breast cancer cell	Cytochalasin D Anti-ICAM-1	The combination of an actin inhibitor and an anti-adhesion molecule antibody inhibited mitochondrial transfer.	184 13
	MSCs / ALL	vincristine (VCR)	Microtubule inhibitors interfered with TNTs formation to reduce mitochondrial translocation.	185
	NK T cells or CD3^+^/CD8^+^ T	L-778123 +α-PD1	Farnesyltransferase and geranylgeranyltransferase 1 inhibitors inhibited TNTs formation and mitochondrial transfer.	14

**Table 3:** Nanotechnological regulation of mitochondrial function in diverse cells

Cell type	Nanotechnology	Drug	Regulating mitochondrial function	Therapeutics strategies	Ref.
Tumor cells	Polymer (SHC4H)	Hydroxychloroquine + Type I photosensitizer (SMNB)	Disrupted mitophagy to amplify oxidative stress.	Chemotherapy PDT	237
	Micelles (A6-dMP-VP)	Oncolytic peptide + Type I photosensitizer (verteporfin)	Induced oxidative stress.	PDT Immunotherapy	238
	Micelles (Mito-CMPN)	Cisplatin + Mitoxantrone	Induced mitochondrial stress.	Chemotherapy PDT	217
	Micelles (CAT/CPT-TPP/PEG-Ce6)	Cinnamaldehyde + Catalase + Photosensitizer (Chlorin e6)	Induced oxidative stress and apoptosis.	PDT Immunotherapy	239
	Semiconduction polymer (TCa/SPN/a)	Ca^2+^ + cGAMP	Induced oxidative stress.	RDT Immunotherapy	236
	Metal NPs (Zn-LDH@Mg)	Ca^2+^ + Mg^2+^	Induced oxidative stress.	MHT Immunotherapy	219
	metal oxide NPs (TiO_2_-_x_F_x_)	TiO_2_-_x_F_x_	Induced mitochondrial stress.	SDT Immunotherapy	30
	Metal oxide NPs (ZnCo-Fe_3_O_4_ CSNCs)	Zn^2+^ + Fe^2+^	Disrupted morphology of mitochondria, depolarization of ΔΨm.	TAE MHT Immunotherapy	220
	Metal NPs (Sb_2_Se_3_@Pt)	Sb_2_Se_3_ + Pt	Induced oxidative stress.	SCT Immunotherapy	221
	Micelles (VB12-sericin-PBLG-IR780)	IR780	Inducing pyroptosis via damaged mtDNA.	PTT PDT Immunotherapy	222
TAMCs	Polymer (mPEI/M1mt)	Mitochondria	Transplanted mitochondria.	Immunotherapy	240
	Dendrimer (D-DPA)	hydroxyl-terminated polyamidoamine (PAMAM) dendrimers	Induced immunosuppressive TAMs apoptosis.	Immunotherapy	224
	Micelles (Man-PEI-PCL/PEG-DMMA)	Mitofusin 1 shRNA (shMFN1) + Celastrol	Repolarized M2-TAMs to M1 phenotype.	Chemotherapy Gene therapy Immunotherapy	225
CAFs	Mesoporous Silica (Se@A&F)	AMD3100	Ruptured mitochondrial structure.	Chemotherapy Immunotherapy	241
	Nanocages (HNav-FAP)	Navitoclax	Induced CAFs apoptosis.	Chemotherapy	242
CSCs	Liposomes (RTB)	β-lapachone (LAP) + pDNA^iNOS^	Decreased mitochondrial OXPHOS.	Radio-immunotherapy Gene therapy	227
	Polymer (CuET@PHF)	CuET nanocrystals	Induced CSCs apoptosis.	Chemotherapy PTP Immunotherapy	243
	Polymer (CuET/ICG)	CuET nanocrystals + ICG	Induced oxidative stress.	Chemotherapy PDT Immunotherapy	232
T cell	Metal NPs (mitoNIDs)	Gold NPs	Induced mitochondria degradation via mitophagy.	Chemotherapy Immunotherapy	233
	Biometic NPs (O-TMV@ABP)	Axitinib + PF-06446846	Promoted T cell mitochondrial biogenesis.	Chemotherapy Immunotherapy	234
	Biometic NPs (PmMN@Om&As)	oxymatrine (Om) + Astragaloside IV (As)	Upregulated T cell biogenesis.	Chemotherapy Immunotherapy	235
	Liposome (L@Mn@SPD)	Mn + Spermidine	Increased mitochondrial respiration.	Chemotherapy Immunotherapy	236
